# The digestive system in Zygentoma as an insect model for high cellulase activity

**DOI:** 10.1371/journal.pone.0212505

**Published:** 2019-02-28

**Authors:** Ratnasri Pothula, Derek Shirley, O. P. Perera, William E. Klingeman, Cris Oppert, Heba M. Y. Abdelgaffar, Brian R. Johnson, Juan Luis Jurat-Fuentes

**Affiliations:** 1 Department of Entomology and Plant Pathology, University of Tennessee, Knoxville, Tennessee, United States of America; 2 USDA-ARS Southern Insect Management Research Unit, Stoneville, Mississippi, United States of America; 3 Department of Plant Sciences, University of Tennessee, Knoxville, Tennessee, United States of America; 4 Department of Entomology and Nematology, University of California, Davis, California, United States of America; USDA Forest Service, UNITED STATES

## Abstract

The digestive system of selected phytophagous insects has been examined as a potential prospecting resource for identification of novel cellulolytic enzymes with potential industrial applications. In contrast to other model species, however, limited detailed information is available that characterizes cellulolytic activity and systems in basal hexapod groups. As part of a screening effort to identify insects with highly active cellulolytic systems, we have for the first time, identified species of Zygentoma that displayed the highest relative cellulase activity levels when compared to all other tested insect groups under the experimental conditions, including model species for cellulolytic systems such as termite and cockroach species in Rhinotermitidae (formerly Isoptera) and Cryptocercidae (formerly Blattodea). The goal of the present study was to provide a morphohistological characterization of cellulose digestion and to identify highly active cellulase enzymes present in digestive fluids of Zygentoma species. Morphohistological characterization supported no relevant differences in the digestive system of firebrat (*Thermobia domestica*) and the gray silverfish (*Ctenolepisma longicaudata*). Quantitative and qualitative cellulase assays identified the foregut as the region with the highest levels of cellulase activity in both *T*. *domestica* and *C*. *longicaudata*. However, *T*. *domestica* was found to have higher endoglucanase, xylanase and pectinase activities compared to *C*. *longicaudata*. Using nano liquid chromatography coupled to tandem mass spectrometry (nanoLC/MS/MS) and a custom gut transcriptome we identified cellulolytic enzymes from digestive fluids of *T*. *domestica*. Among the identified enzymes we report putative endoglucanases matching to insect or arthropod enzymes and glucan endo-1,6-β-glucosidases matching bacterial enzymes. These findings support combined activities of endogenous and symbiont-derived plant cell wall degrading enzymes in lignocellulose digestion in Zygentoma and advance our understanding of cellulose digestion in a primitive insect group.

## Introduction

Although research on cellulolytic systems in insects was initially confined to symbiotic microorganisms [1], in the last decade insect endogenous plant cell wall degrading enzymes (PCWDEs) have been described in Blattodea, Coleoptera, Orthoptera, Phthiraptera, Hemiptera, Phasmida, Lepidoptera, Diptera, and Hymenoptera [2,3–5]. Much of the research on insect cellulolytic enzymes has been concentrated on species of Blattodea, Coleoptera, Lepidoptera and Diptera, probably because some of the insects in these taxa can extensively damage plants and wood, and also due to the availability of sequenced genomes and other metagenomic resources [2,6]. In contrast, cellulolytic systems in other insect orders that contain species specialized to feed on cellulosic materials are understudied.

Enzymatic degradation of cellulose to glucose subunits involves the combined action of three types of enzymes based on their mode of action and substrate specificities. Endoglucanases (EC 3.2.1.4) cut at random internal points in cellulose chains, while cellobiohydrolases (EC 3.2.1.91) cleave at the non-reducing ends releasing cellobiose units that are digested to glucose by β-glucosidases (EC 3.2.1.21) [2].

As part of a screening effort to identify insects with highly active cellulolytic systems [7], we detected species of Zygentoma that displayed the highest relative cellulase activity levels against carboxymethylcellulose (CMC) compared to all other tested insect orders. Members of Zygentoma are known to feed and digest highly cellulosic materials such as paper, cardboard, flour, and insulation [8,9]. Digestive system descriptions for *Thermobia aegyptiaca* and *Lepisma saccharina* supported similarities with Orthoptera, including being slightly longer than body length, differentiated into foregut, midgut and hindgut regions, and containing a muscular proventriculus with sclerotized teeth like structures [10,11]. Production of endogenous cellulases was previously reported in *Ctenolepisma lineata* and firebrat (*Thermobia domestica*) [12–14]. Additionally, cellulase activity was localized mostly to the crop in *T*. *domestica* [12]. More recently, Sabbadin et al [15] investigated the digestive proteome of *T*. *domestica* and identified carbohydrate-degrading enzymes, including lytic polysaccharide monooxygenases (LPMOs), which weaken cellulose fibers making them more accessible to cellulose degradation.

The goal of the present study was to provide a morphohistological characterization of cellulose digestion and to identify enzymes responsible for high cellulase activity in digestive fluids of Zygentoma. Initial characterization supported no relevant morphological differences in the digestive tube of the firebrat (*T*. *domestica*) and the gray silverfish (*Ctenolepisma longicaudata*). Quantitative and qualitative cellulase assays confirmed the foregut as the region with the highest cellulase activity, and *T*. *domestica* as displaying higher endoglucanase, xylanase and polygalacturonase activities compared to *C*. *longicaudata*. Using a custom gut transcriptome and nano liquid chromatography tandem mass spectrometry analysis (nanoLC/MS/MS) we report the identification of predicted endogenous and symbiont-derived PCWDEs from partially purified digestive extracts from *T*. *domestica*. These findings advance our understanding of cellulose digestion in a basal hexapod group.

## Materials and methods

### Insects

Nymphs and adults of silverfish (*Ctenolepisma longicaudata*) were hand-collected in buildings using open plastic dishes (15 cm) (Pioneer Plastics Inc., Dixon, KY) baited with whole-grain oat flakes (Quick 1-Minute Oats, Quaker Oats Co., Chicago, IL) or swept into collection containers using a 3” long, soft-bristle make-up brush. Collected specimens were reared in the laboratory at room temperature on rolled oats, paper, and dry dog food (Pedigree Adult Complete Nutrition, Mars, Inc., Mount Olive, NJ) as protein source.

An established culture of firebrat (*Thermobia domestica*) was generously provided by Patrick Stanley and Eric Snell (Snell Scientific LLC, Meansville, GA) from a colony derived from a culture at the Department of Entomology at Ohio State University (Columbus, OH). This colony has been maintained at 34°C in a dark incubator in the Department of Entomology and Plant Pathology at the University of Tennessee for >8 years using printer paper and NatureWise chick starter grower feed (Nutrena, Minneapolis, MN) as carbohydrate and protein source, respectively.

### Gut morphology and histology

For gut morphological studies, adult *C*. *longicaudata* and *T*. *domestica* were anesthetized for 10 min at 4°C and dissected under a Zeiss Stemi 2000-C stereo microscope (Carl Zeiss Microscopy, LLC, Thornwood, NY). The gut was carefully dissected from the rest of the body and images were taken with a Canon DS126311 camera (Canon, Ota, Tokyo, Japan) mounted on the stereo microscope. Adult *C*. *longicaudata* and *T*. *domestica* for histological studies were sacrificed by incubation at -20°C for ten minutes and then fixed in Carnoy’s solution (60% ethanol, 30% chloroform, and 10% glacial acetic acid) for four hours at 4°C. After fixing, whole insects were transferred to 70% ethyl alcohol and sent to the Biomedical and Diagnostic Services, University of Tennessee College of Veterinary Medicine (Knoxville, TN) for sectioning and staining with hematoxylin and eosin. Histological sections were examined and documented using an Olympus BX63F upright microscope (Olympus Corporation, Shinjuku, Tokyo, Japan).

### Preparation of samples for biochemical tests

Individual, adult *C*. *longicaudata* and *T*. *domestica* were starved in Petri dishes for five days, and then fed with high cellulose diet represented by standard 92 multipurpose printing paper (Georgia-Pacific, Atlanta, GA, USA), or control (bovine serum albumin) diet, and allowed to feed for five days under culture conditions. A piece of 1% agar was provided and changed every other day as a water source. On the 11^th^ day, anesthesia and dissections were carried out on ice. The digestive tract along with the head were separated from the rest of the body, and the gut was further divided into the foregut, midgut ad hindgut regions using a sterile scalpel. The head was separated from the foregut, and the midgut region was identified from the origin of gastric caeca to the origin of Malpighian tubules, which was considered as the start for the hindgut region. Tissues pooled from six individual insects were placed in microfuge tubes containing 100 μl of sterile water and the digestive fluids were extracted by gently pressing the tissues against the tube wall with disposable pellet pestles, briefly vortexing and then centrifuging at 21,000 x *g* for 3 min at room temperature. The supernatant was collected into microfuge tubes as the digestive fluid sample and stored at -80°C until used.

### Zymography

Cellulase activity in tissues of *C*. *longicaudata* and *T*. *domestica* was tested using zymography on SDS-12%PAGE gels containing 0.2% carboxymethyl cellulose (CMC) as substrate [16]. The sample fluids were allowed to thaw on ice and protein concentrations were estimated using the Protein Quantification kit in a Qubit fluorometer (Invitrogen, Carlsbad, CA). Samples (100 μg) were mixed with an equal volume of 2X sample buffer (50 mM Tris–HCl, pH 6.8, 2% SDS, 10% glycerol, 1% β-mercaptoethanol, 0.01% bromophenol blue) and the mixture was partially denatured by heating at 72°C for 15 min. Commercial endoglucanase from *Aspergillus niger* (Tokyo Chemical Industry Co. Ltd., Portland, OR) was used as positive control. Samples were resolved by electrophoresis at 100 V until the dye reached the bottom of the gel and the gels were washed in 0.1 M sodium succinate buffer (pH 5.8) containing 10 mM dithiothreitol (DTT) for five washes of 30 min with constant shaking. Gels were then incubated in 0.1 M sodium succinate buffer (pH 5.8) with no DTT for 30 min at 60°C and then stained with 0.1% of Congo red (Acros Organics, Waltham, MA) for 10 min. Gels were destained by incubating in 1 M NaCl until the cellulase activity bands were visible as clear bands on a red background. Glacial acetic acid (2 μl/ml) was added to shift the background gel color to dark-purple for more clear observation of activity bands. Gel images were taken with a Versadoc 1000 Imager (Bio-Rad, Hercules, CA).

### Quantification of cellulase, xylanase and polygalacturonase activities

**Fig 1:**
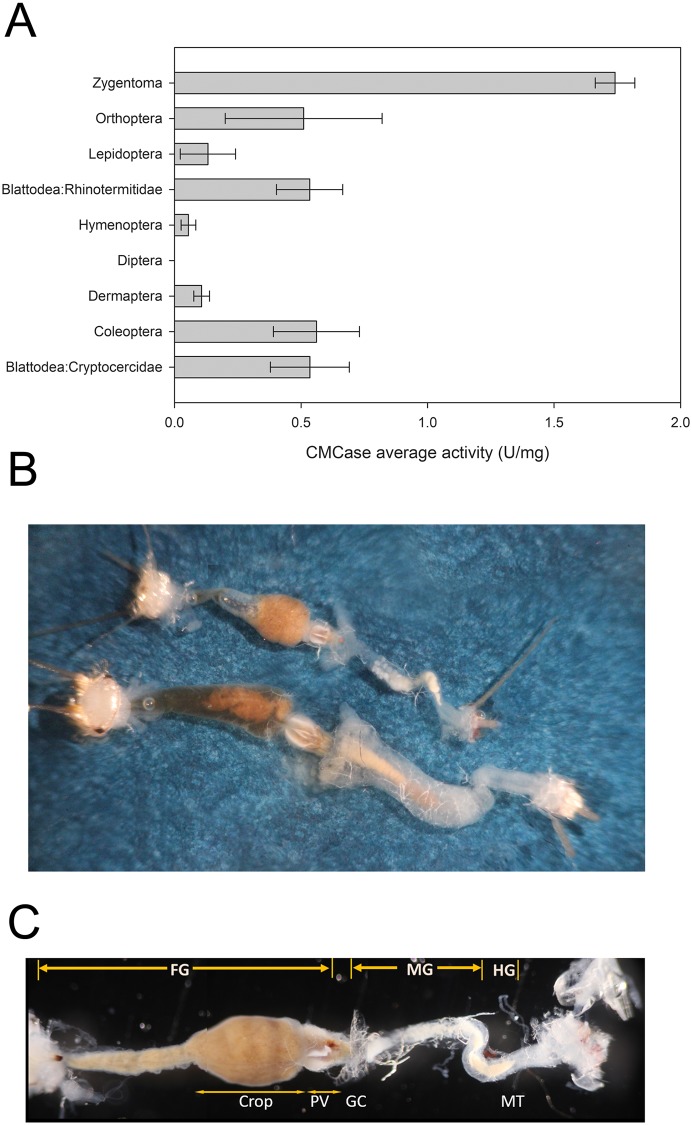
Structural comparison of the digestive tract in firebrat (*Thermobia domestica*) and silverfish (*Ctenolepisma longicaudata*), and relative CMCase activity in Zygentoma compared to other insect orders. A) Activity (U/mg) of gut digestive fluids against CMC substrate in the most active samples from species of Zygentoma (*C*. *longicaudata*), and representative species from Orthoptera (*Conocephalus strictus*), Lepidoptera (*Halysidota tessellaris*), Blattodea (formerly Isoptera): Rhinotermitidae (*Reticulitermes flavipes*), Hymenoptera (*Neodiprion lecontei*), Diptera (*Monarthropalpus flavus*), Dermaptera (*Forficula auricularia*), Coleoptera (*Scolytinae* spp.) and Blattodea (formerly Blattaria): Cryptocercidae (*Cryptocercus* spp.). Shown are the average activity and corresponding standard error from at least three biological replicates performed in triplicate for each species. All experiments were concurrent, but all activities except for the Zygentoma sample were taken from Oppert *et al*. [7]. Dissected digestive tracts of firebrat (top) and silverfish (bottom). Note the relatively larger size of the tract in silverfish compared to firebrat. C) Morphological parts of the digestive tract of firebrat. FG, foregut and crop; MG, midgut; HG, hindgut; PV, proventriculus; GC, gastric caecae; MT, Malpighian tubules.

Quantitative activity against carboxymethylcellulose (CMC) in gut fluids of diverse insects (shown in Fig 1A) was determined as described in Oppert et al. (2010). Assays with gut fluids from *C*. *longicaudata* were performed concomitantly but were not originally included in Oppert et al. (2010).

In the present study, the protein content in dissected head and foregut samples was quantified using the Qubit Protein Quantification kit in a Qubit fluorometer (Invitrogen, Carlsbad, CA). Cellulose degrading activity in head and foregut tissues of *C*. *longicaudata* and *T*. *domestica* was quantified using a cellulase assay kit (Megazyme, Ireland) to quantify endoglucanase activity against 4-nitrophenyl-β-D-cellopentaoside (BPNPG5) as substrate, 4-nitrophenyl β-D-cellobioside (pNPC) (Sigma-Aldrich, St. Louis, MO) as substrate for β-glucosidase, 4-nitrophenyl β-D-xylopyranoside (pNPX) (Sigma-Aldrich, St. Louis, MO) as substrate to quantify β-xylosidase activity, and pectin from citrus peel (Sigma-Aldrich, St. Louis, MO) to measure polygalacturonase activity.

Endoglucanase activity was measured in samples (5 μg of protein in 25 μl) mixed with 50 μl of substrate and incubated at 40°C for 10 min. Reactions were terminated by adding alkaline solution (125 μl of Tris buffer solution, pH 9.0) and absorbance was measured at 405 nm in a Synergy HT microplate reader (BioTek, Winooski, VT) using the Gen5 software (v. 2.0, BioTek, Winooski, VT). CellG5 activity was calculated according to the Mega-Calc method from the manufacturer (Megazyme, Leinster, Ireland). One unit of cellG5 activity is defined as the amount of endoglucanase required to release 4-nitrophenol from BPNPG5 in one minute at 40°C in the presence of excess thermostable β-glucosidase.

Both β-glucosidase and β-xylosidase activities were measured in samples (20 μl containing 10 μg of protein) mixed with 130 μl of 10 mM substrate in 50 mM sodium acetate buffer (pH 5.0) and incubated at 50°C for 30 min. Reactions were stopped by addition of 1 volume of 2 M Na_2_CO_3_ and absorbance was measured at 405 nm as described above. A 4-nitrophenol standard curve (0–1 mM) was used to quantify specific activity, and background product in reactions was corrected by subtracting final from initial product amounts. Specific activity was expressed in U/mg of protein, with 1 U defined as the amount of enzyme resulting in production of 1 μmol of 4-nitrophenol per min at pH 5.0 and 50°C.

Polygalacturonase activity was determined in samples (10 μg of protein in 20 μl) mixed with 15 μl of 1% pectin and 115 μl of 50 mM sodium acetate buffer (pH 5.0). The mixture was incubated at 50°C for 1 h, and then 50 μl of 3,5-dinitrosalicylic acid (DNSA) reagent was added and absorbance measured at 540 nm as above. The specific activity was calculated using a glucose standard curve (0–20 mM) and background glucose was corrected by subtracting final from initial values. Specific activity was expressed in U/mg of protein, with 1 U defined as the amount of enzyme resulting in production of 1 μmol of glucose per min at pH 5.0 and 50°C.

All activity assays were carried out using at least three biological samples tested in reactions performed in triplicate. The statistical design for each activity assay was a completely randomized design with a 2x2x2 factorial. Statistical analyses were performed through SAS (SAS Institute, Inc., Cary, NC) using mixed model analysis of variance. Prior to analysis, data that failed to pass the Shapiro-Wilk normality test were log transformed. Least square means were separated using Tukey’s option and significant differences were considered at *P* < 0.05.

### Cellulase enzyme purification

Cellulase enzymes were partially purified from *T*. *domestica* gut fluid extracts using size-exclusion chromatography as follows. Gut fluids (300 μL) were collected from dissected guts as described above and diluted with 0.05 M sodium phosphate buffer at pH 6.0 (Buffer A) to a final volume of 1.5 ml. The sample was centrifuged at 10,000 x *g* for 10 min at room temperature and supernatant was collected and used as enzyme extract. A HiLoad 16/60 Superdex 200 column connected to an AKTA FPLC system (both from GE Healthcare, Chicago, IL) pre-equilibrated with buffer A. The digestive extract was clarified by passing through a 0.22 μm syringe filter before loading on to the column. Proteins were eluted from the column in 2 ml fractions in a total volume of 240 ml of Buffer A at a constant flow rate of 1 ml/min. Cellulase activity in aliquots (20 μl) of the eluted fractions was tested using the CMC-agar plate method [17]. Clear zones in the CMC-plate indicated presence of cellulase activity in the active fractions, which were used for subsequent protein identification.

### Sequencing of custom *T*. *domestica* gut transcriptome

Total RNA was purified from a pool of 10 dissected *T*. *domestica* adult guts preserved in RNAlater (Sigma-Aldrich, St. Louis, MO) using RNAzol (Molecular Research Center, Cincinnati, OH), following the manufacturer’s instructions. Purified samples were then processed using an RNAeasy kit (Qiagen, Hilden, Germany) to eliminate contaminants before library preparation. Total RNA was then digested with 5 units of DNase I at 37°C for 10 min to remove any contaminating genomic DNA followed by incubation at 75°C for 10 min to inactivate DNase I. Messenger RNA (mRNA) was enriched from the total RNA using PolyA+ Tract reagents (Promega, Madison, WI) following manufacturer’s instructions. Briefly, total RNA was mixed with 150 pmol of biotin labeled oligo-dT_(20)_ 2X SSC and annealed to the mRNA by heating to 75°C 5 min followed by cooling slowly to room temperature on a bench top. Streptavidin coated paramagnetic beads supplied with the kit were washed three times with 2X SSC buffer, resuspended in 100 μl of 2X SSC, and mixed with RNA annealed to biotin-Oligo-dT. After 10 min incubation at room temperature, a magnetic stand was used to collect mRNA bound to magnetic beads. Liquid from the tube was removed with a pipette and the paramagnetic beads were washed with 0.5X SSC three times. RNase-free DEPC treated water (100 μl) was used to elute the mRNA, which was transferred to a new tube after collecting the paramagnetic beads on a magnetic stand.

The SuperScript Double-Stranded cDNA Synthesis Kit (Invitrogen, Carlsbad, CA) was used to synthesize cDNA from 100 ng of mRNA following manufacturer’s instructions. The double-stranded cDNA reaction was adjusted to 200 μl with 50 μl of distilled water and purified using 200 μl of AmpureXP magnetic beads (Beckman Coulter, Bria, CA) and eluted in 20 μl of 10 mM Tris-HCl. Sequencing libraries were prepared from 10 ng of cDNA using the Illumina Nextera library construction kit (Illumina, San Diego, CA). The cDNA libraries were size-fractionated on a Pippin Prep instrument (Sage Science Inc., Beverly, MA) to obtain 450 ± 20 bp fragments and quantified using the KAPA library quantification kit (KAPA Biosystems, Wilmington, MA). The library was sequenced on an Illumina Hiseq2500 instrument at the USDA-ARS Genomics and Bioinformatics Research Unit (Stoneville, MS).

Raw reads were trimmed and *de novo* assembled in SeqMan NGen 3.1.1 build 15 in the DNASTAR software (Madison, WI). Assembly parameters included 21 bp match size, 50 bp match spacing, 93 minimum match percentage, 10 match score, 20 mismatch penalty, 30 gap penalty, and 6 bp maximum gap. From the almost 10 million raw sequence reads analyzed, 6,894,710 (69%) sequences were assembled into 4,558 contigs (average coverage 13X), with 227 contigs >2,000 bp in length and 1,137 bp as contig N50. Contigs were annotated using Blast2Go [18] and the resulting FASTA file used for protein identifications as described below. The raw reads and assembled transcriptome were deposited in the sequence read archive (SRA) database at NCBI with accession number SRX4721443. The Transcriptome Shotgun Assembly (TSA) project has been deposited at DDBJ/EMBL/GenBank under the accession GHEH00000000. The version described in this paper is the first version, GHEH01000000.

### Identification of cellulases using nanoLC/MS/MS

Fractions from the size exclusion purification (20 μg) assays that yielded CMCase activity were resolved in replicated SDS-10% PAGE gels. One of the gels was stained for total protein (ProtoBlue Safe stain, National Diagnostics, Atlanta, GA) and the replica gel used for zymography with CMC as substrate, as explained above. Cellulase active protein bands in the zymogram were excised from the stained gel by comparing the gels and the position of prestained molecular markers. Excised bands (3) were sent to MS Bioworks LLC (Ann Arbor, MI) for nano liquid chromatography tandem mass spectrometry analysis (nanoLC/MS/MS). Gel pieces were washed with 25 mM ammonium bicarbonate followed by acetonitrile, and then reduced with 10 mM DTT at 60°C and alkylated with 50 mM iodoacetamide at room temperature. Proteins were subjected to trypsin digestion for 4 h at 37°C and then quenched with formic acid. The supernatant was analyzed directly by nanoLC/MS/MS with a Waters NanoAcquity HPLC system interfaced to a LTQ Orbitrap Velos mass spectrometer (Thermo Fisher Scientific, Waltham, MA). Peptides were loaded on a trapping column packed with Jupiter Proteo resin (Phenomenex, Torrance, CA) and eluted over a 75 μm analytical column at 350 nL/min. The mass spectrometer was operated in data-dependent mode, with MS performed in the Orbitrap at 60,000 FWHM resolution and MS/MS performed in the LTQ. The fifteen most abundant ions were selected for MS/MS. Data were queried against the custom *T*. *domestica* transcriptome using the Mascot software (Matrix Science, London) with carbamidomethyl (C) as fixed modification, and oxidation (M), acetyl (protein N-terminus), and deamidation (NQ) as variable modifications. Additional parameters included 10 ppm peptide mass tolerance, 0.02 Da fragment mass tolerance and two maximum missed cleavages. Mascot DAT files were parsed into the Scaffold v. 4.8.7 (Proteome Software Inc., Portland, OR) for validation, filtering, and to create a non-redundant list per sample. Data were filtered using a minimum protein value of 99%, a minimum peptide value of 95.5% (Prophet scores) and requiring at least two unique peptides per protein. The list of identified proteins was organized based on protein abundance using the Normalized Spectral Abundance Factor (NASF) quantitative method [19], which takes into account the normalized spectra and protein length. The transcriptome contigs matching to identified proteins are annotated for GH family in CAZy database and the matching sequences are presented in S1 Table.

## Results

### High cellulase activity and gut morphology in Zygentoma

As a part of a quantitative prospecting effort to identify insects with high cellulolytic activity [7], we detected *C*. *longicaudata* as having the highest relative cellulase (endoglucanase, CMCase) activity among all taxonomic orders tested (Fig 1A). Based on this observation, we focused on species of Zygentoma for characterization of a highly cellulolytic system.

The digestive systems of *T*. *domestica* and *C*. *longicaudata* had similar morphology and histology, although the digestive tube in *C*. *longicaudata* was relatively longer and larger than in *T*. *domestica* (Fig 1B). Consequently, we focused on *C*. *longicaudata* for further characterization of the digestive tube due to its relatively larger size. This digestive tube was longer than the insect body length and could be divided into foregut, midgut, and hindgut regions. The foregut was the largest part of the digestive system and included an enlarged crop extending throughout the thoracic region and making up half of the digestive tube (Fig 1C). In several dissections and inspections of the three gut compartments, a food bolus was observed within the crop region of the foregut.

**Fig 2:**
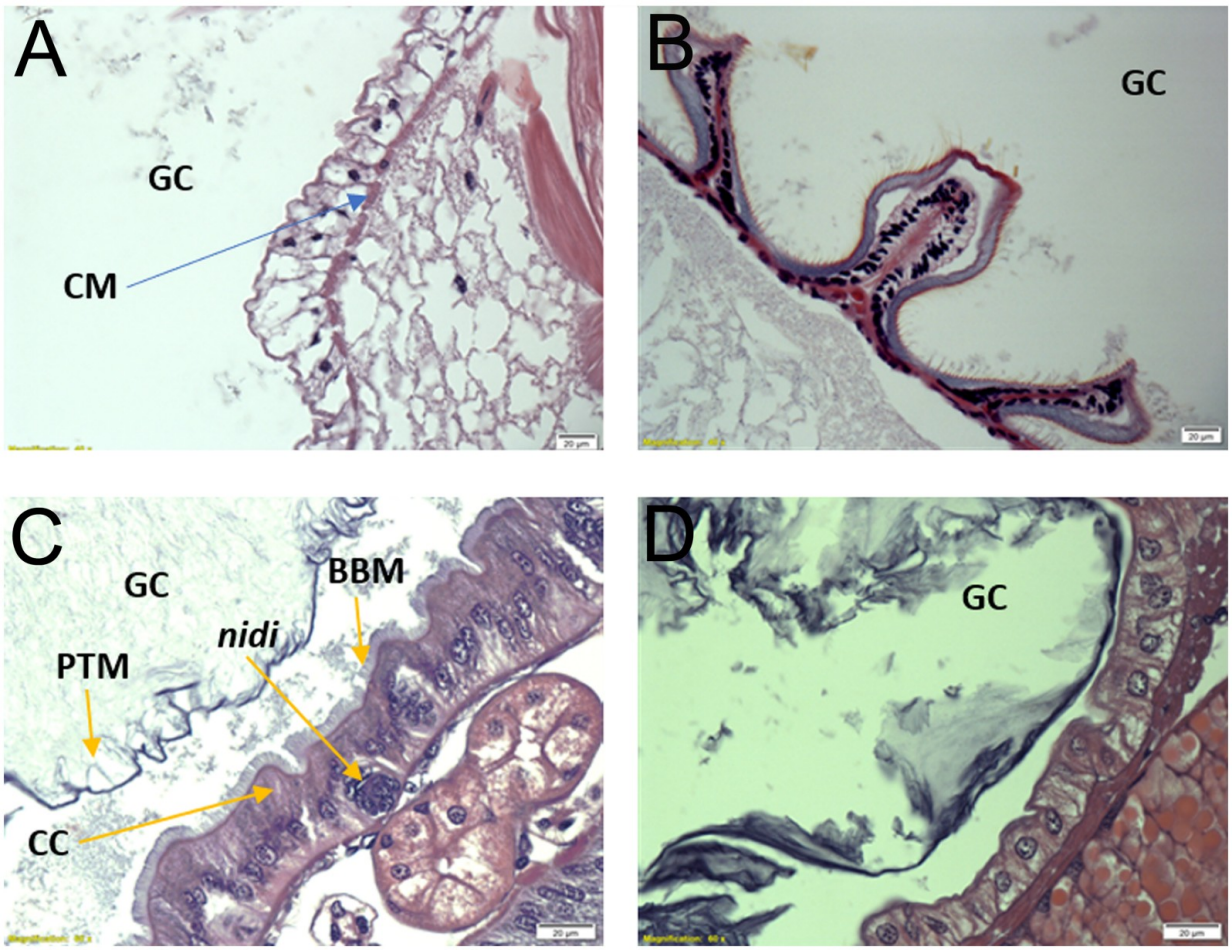
Histology of the digestive system regions in *Ctenolepisma longicaudata*. A) Longitudinal section of the crop showing the monolayer of epidermal cells and the underlying circular muscle cells. B) Longitudinal section of proventriculus (PV). C) Longitudinal section of midgut showing peritrophic membrane, columnar cells lined with brush border membrane and intermitted by a group of nidi cells at the bottom. D) Longitudinal section of hindgut wall showing the monolayer of epidermal cells. All sections were stained with hematoxylin and eosin stain. GC, gut cavity; CM, circular muscle cells; PTM, peritrophic matrix; CC, columnar cells; BBM, brush border membrane.

Histological observations confirmed previous drawing descriptions of the gross morphology and cell types present in the digestive tissue of Zygentoma [10]. The crop wall in *C*. *longicaudata* consisted of a monolayer of epidermal cells supported by circular muscle cells (Fig 2A). The crop opened posteriorly into the proventriculus, which was highly muscular and had six sclerotized teeth-like structures (Fig 2B). The midgut was the second longest part of digestive system and appeared as a simple tube-like structure with gastric caecae at the anterior region. The midgut epithelium (Fig 2C) was characterized by the presence of a single layer of columnar cells with apical brush border membrane, and nidi of stem cells appeared interspersed in the epithelium. The connection between midgut and hindgut was traced by the presence of Malpighian tubules, which were numerous in number and longer than the insect body length (Fig 1B). The hindgut was a short simple tube-like structure with a monolayer of epidermal cells and ended in rectal pads (Fig 2D).

### Qualitative and quantitative location of cellulase activity in the digestive system of *T*. *domestica* and *C*. *longicaudata*

**Fig 3:**
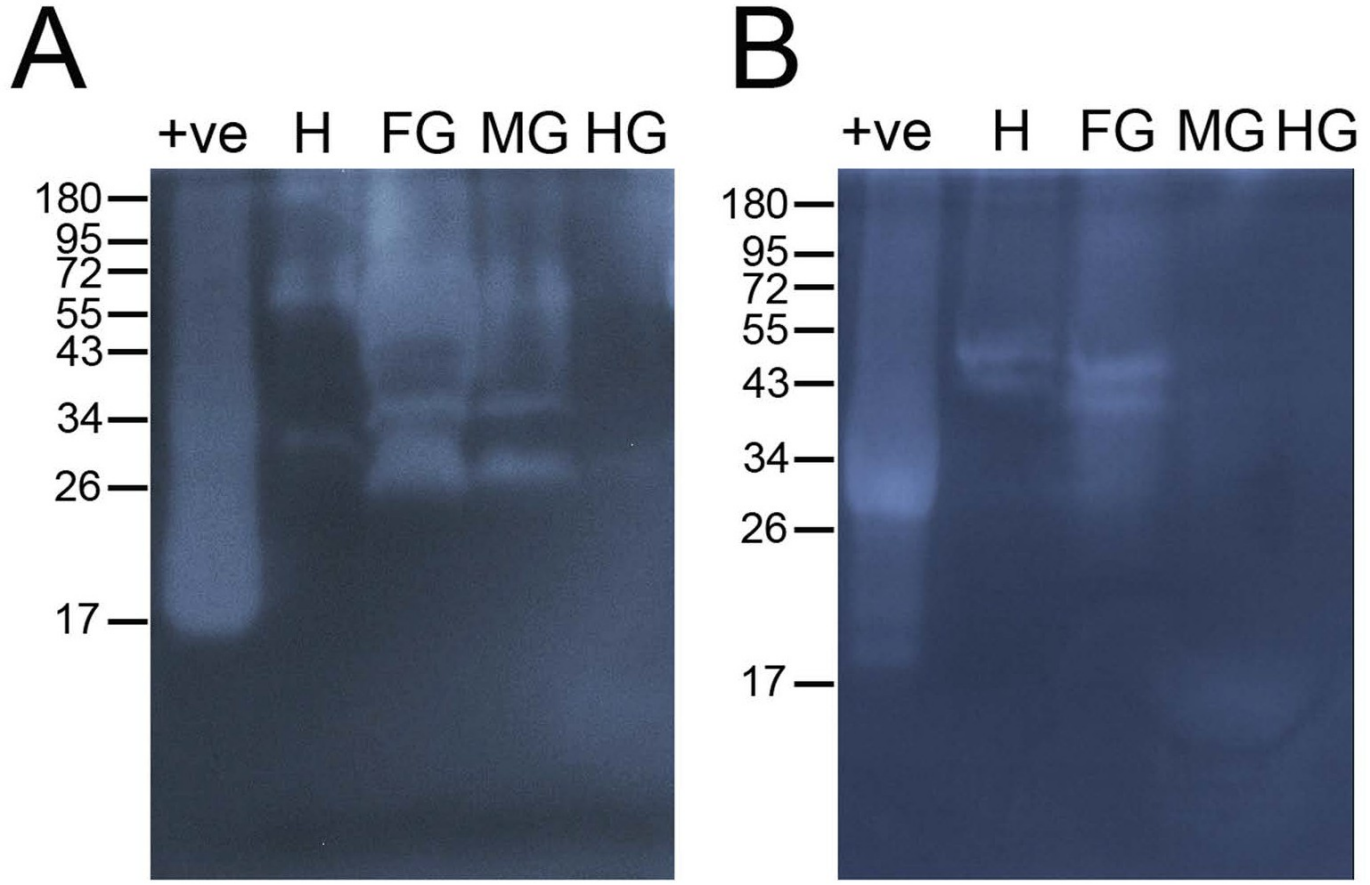
Detection of cellulase activity in digestive fluids of *Thermobia domestica* and *Ctenolepisma longicaudata*. Zymograms with 0.2% carboxymethyl cellulose were used to detect the cellulase (CMCase) activity in head, foregut, midgut and hindgut tissues of *T*. *domestica* (left) and *C*. *longicaudata* (right). MM, pre-stained protein molecular marker; +ve, commercial cellulase used as positive control; H, head; FG, foregut; MG, midgut; HG, hindgut.

Zymograms of *T*. *domestica* gut samples had more and brighter bands of activity against CMC compared to *C*. *longicaudata* (Fig 3). When comparing among gut regions in both species, higher cellulase activity was found in samples from the head and foregut compared to midgut and hindgut tissues (Fig 3). Consequently, head and foregut tissues were selected for quantitative detection of plant cell wall degrading enzymes (PCWDEs).

**Fig 4:**
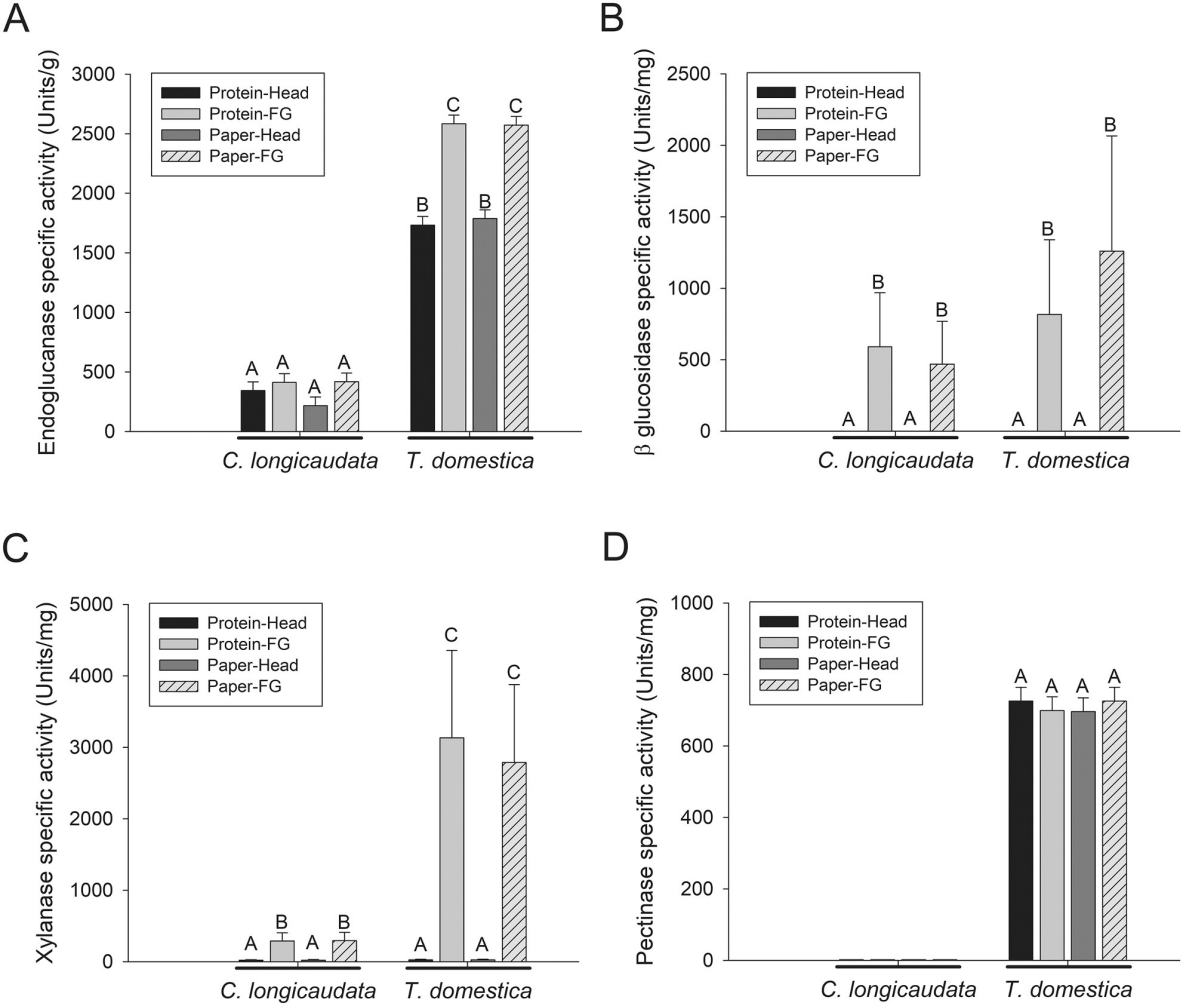
Quantification of plant cell wall degrading enzyme activities in the fluids derived from head and foregut tissues of *Ctenolepisma longicaudata* and *Thermobia domestica*. Fluids from head and foregut (FG) tissues of *C*. *longicaudata* and *T*. *domestica* fed on protein (BSA) or paper diet (see Materials and methods) were used in assays to detect A) endoglucanase activity against 4-nitrophenyl-β-D-cellopentaoside (BPNPG5), B) β-glucosidase activity against 4-nitrophenyl β-D-cellobioside (pNPC), C) β-xylosidase activity against 4-nitrophenyl β-D-xylpyranoside (pNPX), and D) polygalacturonase activity against pectin from citrus peel. Shown are the means and corresponding standard errors calculated from three biological and three technical replicates. Different letters above the bars indicate significant differences in the mean activity (*P* < 0.05). Units of specific enzyme activity are per mg of protein in all the graphs except in graph A) where it is expressed per g of protein. One unit of enzyme activity was defined as the amount of enzyme required to release 1 μmol of 4-nitrophenol from the respective substrate in all the graphs except in D), where it is 1 μmol of glucose.

Enzymatic activities tested quantitatively in digestive fluids obtained from head and foregut samples of both *T*. *domestica* and *C*. *longicaudata* included endoglucanase, β-glucosidase, β-xylosidase and polygalacturonase (Fig 4). As observed in the qualitative zymograms, digestive fluids from both head and foregut tissues of *T*. *domestica* had significantly higher endoglucanase activity compared to *C*. *longicaudata* (*P* < 0.05). Within *T*. *domestica*, the digestive fluids from foregut had significantly higher endoglucanase activity than fluids from head tissue (*P* < 0.05), while significant differences were not observed between samples from foregut and head tissues of *C*. *longicaudata* (Fig 4A). Both *T*. *domestica* and *C*. *longicaudata* had no β-glucosidase activity in head fluids, however similar levels of β-glucosidase activity were found in the digestive fluids from foregut tissues of both species (Fig 4B). β-xylosidase activity was significantly higher (about six-fold greater, *P* < 0.05) in the foregut fluids of *T*. *domestica* compared to *C*. *longicaudata*, and very small levels of β-xylosidase activity were detected in head fluids from both insects (Fig 4C). Polygalacturonase activity was absent from *C*. *longicaudata* and present in both head and foregut tissues of *T*. *domestica* (Fig 4D). Feeding both insects on a protein-rich (BSA) or a cellulose-rich (paper) diet did not result in significant differences in any of the tested enzyme activities (*P* > 0.05) (Fig 4).

### Identification of PCWDEs from digestive fluids of *T*. *domestica*

**Fig 5:**
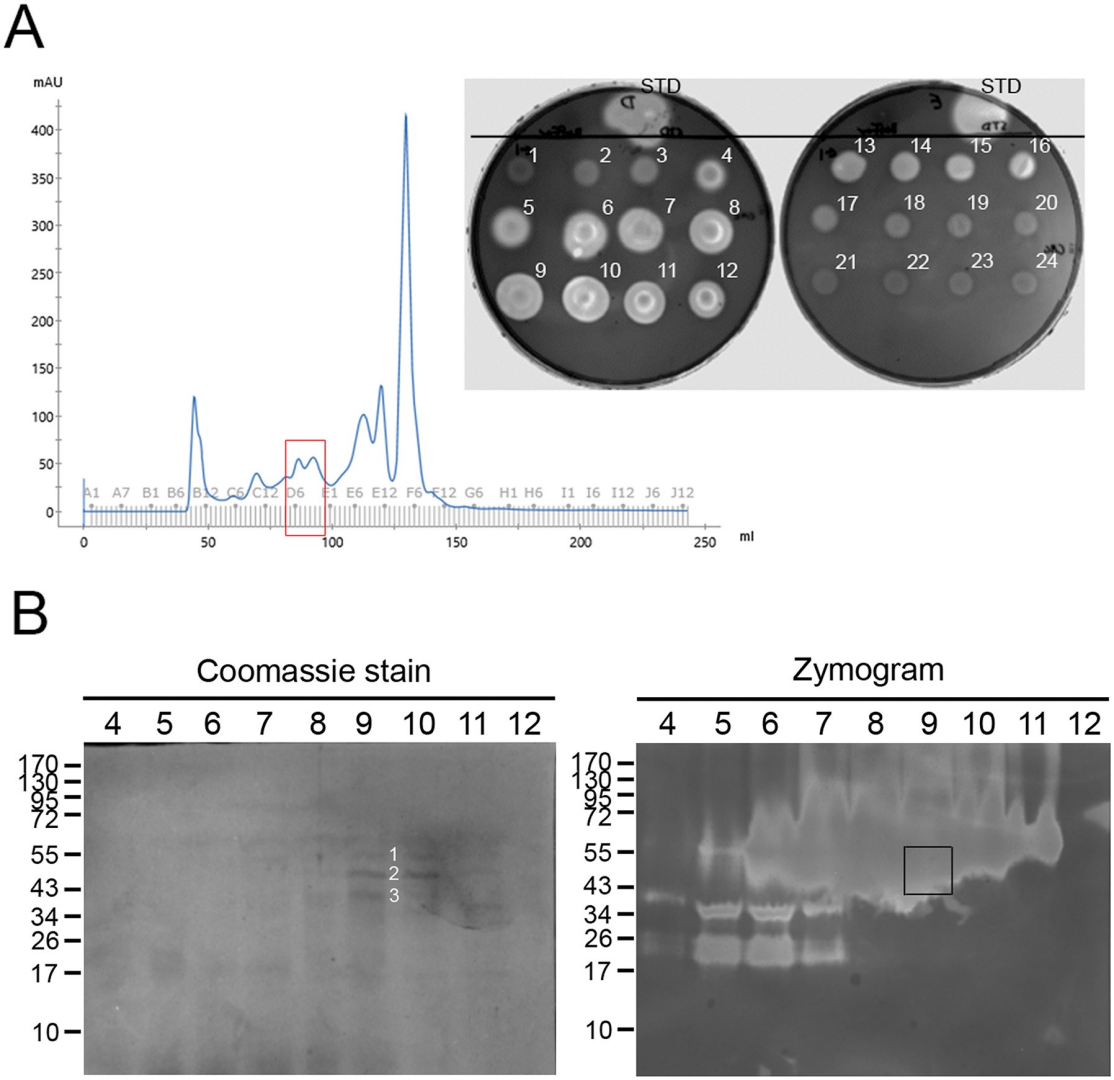
Partial purification of cellulase enzymes from gut fluids of *T*. *domestica* for proteomic analysis. A) Chromatogram from fractionation of gut fluids from the whole digestive system of *T*. *domestica* adults using size exclusion chromatography, and testing for CMCase activity in aliquots from eluted fractions using an agarose plate assay. Consecutively eluted fractions are numbered in the plate, as well as endoglucanase from *A*. *niger* as CMCase positive control (STD). Fractions displaying relatively high levels of CMCase activity (fractions 4–12) were resolved by electrophoresis in semi-denaturing SDS-10%PAGE (B). After electrophoresis, gels were stained for total protein with Coomassie stain or used for zymography with CMC as substrate, as indicated in the figure panels. Three protein bands in the Comassie stained gel replica (denoted by white 1–3 numbers in the figure) that approximately corresponded to the region including CMCase activity in the zymogram (delimited in the zymogram with a black square) were cut from the stained gel and submitted for mass spectrometry.

Considering the higher cellulase activity of *T*. *domestica* compared to *C*. *longicaudata*, and the large amount of material needed for purification and proteomic analysis, we focused on the partial purification and identification of cellulases from digestive fluids obtained from the whole digestive tube of *T*. *domestica*. After size-exclusion chromatographic purification, nine fractions were identified as containing relevant endoglucanase (CMCase) activity based on agar plate assays (Fig 5A). These samples were resolved by electrophoresis and protein staining and zymography to detect protein bands located in areas of high CMCase activity in the zymogram (Fig 5B). Fraction D9 had the most clearly visible protein bands when stained with Coomassie, and these bands were located in an area with high levels of CMCase activity in the zymogram. Consequently, the three protein bands clearly observed in this fraction were selected and excised as band 1 (approx. 55 kDa), band 2 (approx. 49 kDa), and band 3 (approx. 42 kDa) for mass spectrometry analysis. Activity against CMC in the 42–55 kDa region was also detected in zymograms of the firebrat foregut and midgut (Fig 3).

The excised bands were subjected to nanoLC/MS/MS for protein identification using a custom *T*. *domestica* gut transcriptome for database searches, which detected a total of 21 proteins (Table 1) of diverse Normalized Spectral Abundance Factor (NSAF). Among the identified proteins, there were a majority of enzymes involved in digestion of carbohydrates and their polymers (endoglucanase, α-amylase, endo-β-1,4-mannanase, β-galactosidase) and few enzymes involved in chitin metabolism (chitinase, chitin deacetylase). Most contigs identified matched to insect proteins, except for four contigs matching to proteins from crustaceans (two chitinases, an endo-β-1,4-mannanase and an endoglucanase) and two bacterial glucan endo-1,6- β-glucosidases (Table 1). Overall, endoglucanases were the most common (9 proteins identified) and relatively abundant, as they represented 41.5%, 85.1%, and 76.5% of the NSAF from bands 1, 2, and 3, respectively. Among the endoglucanases, the most abundant protein matched to contig23 in the *T*. *domestica* transcriptome, which made up >40% of the endoglucanase NSAF in each band. Only in band 1 was an α-amylase matching to contig62 was more abundant than contig23. The second group of most abundant endoglucanases matched to contig56, contig253, contig309, contig50, and contig9 (in decreasing order of NSAF). Other proteins detected with relevant NSAF were a luciferin-regenerating enzyme matching to contig359, a chymotrypsin 1 matching to contig5, and endo-β-1,4-mannanases matching to contig25 and contig4.

**Table 1:** Proteins identified by nanoLC/MS/MS in fractionated gut fluids from *Thermobia domestica* with CMCase activity. The contigs identified in the transcriptome and the matching proteins in the NCBInr database are listed, as well as the normalized quantitative values NSAF in each of the electrophoretic bands analyzed, and the respective glycoside hydrolase family assigned by the CAZy database.

Contig	Identified protein	Organism	E-Value	Similarity	Accession number	Band 1 NSAF	Band 2 NSAF	Band 3 NSAF	GH family
23, 309	endoglucanase	*Mastotermes darwiniensis*	0	79.9%	CAD54729.1	0.33983	0.39581	0.33043	GH9
62	α-amylase	*Blattella germanica*	0	78.2%	ABC68516.1	0.44423	0.06650	0.10558	GH13
56	endoglucanase	*Panesthia cribrata*	0	75.4%	AAF80584.1	0.01421	0.09824	0.12462	GH9
359, 444	Similar to luciferin-regenerating enzyme	*Tribolium castaneum*	2.06e-91	68.0%	XP_967986.1	0.05541	0.02064	0.01893	NA
50, 11	endoglucanase	*Coptotermes acinaciformis*	0	72.8%	AAK12339.1	0.01130	0.05306	0.02849	GH9
9	endoglucanase	*Coptotermes formosanus*	0	77.2%	BAB40697.1	0	0.02094	0.02377	GH9
253	endoglucanase	*Coptotermes formosanus*	8.38e-17	81.8%	ADB12483.1	0	0.24373	0.17412	GH9
5	Chymotrypsin	*Teleogryllus emma*	3.69e-76	67.6%	ABV32556.1	0.02379	0.02740	0.02474	NA
4, 25, 488	Endo-β-1,4-mannanase	*Daphnia pulex*	1.23e-132	68.1%	EFX71596.1	0.02143	0.01737	0.04125	GH5
61	endo-1,3(4)-β-glucanase	*Periplaneta americana*	1.42e-135	71.1%	ABR28480.1	0	0	0.022158	GH16
65	Acidic mammalian chitinase	*Daphnia pulex*	1.24e-113	66.4%	EFX90412.1	0.01220	0	0.01097	GH18
234	endoglucanase	*Mastotermes darwiniensis*	3.76e-39	76.9%	AAF63725.1	0.05048	0.03875	0.03999	GH9
1026	Similar to β-galactosidase	*Tribolium castaneum*	0	64.6%	XP_967647.1	0.00798	0	0	GH35
68, 54	Chitinase	*Marsupenaeus japonicus*	1.68e-104	63.0%	BAA12287.1	0.01909	0	0	GH18
157	Hypothetical protein SINV_01527	*Solenopsis invicta*	3.78e-162	65.3%	EFZ09833.1	0	0	0.010978	GH18
240	endoglucanase	*Daphnia pulex*	1.00e-13	76.8%	EFX80604.1	0	0	0.04438	GH9
383	Chitin deacetylase 9	*Tribolium castaneum*	1.40e-102	59.8%	ABW74152.1	0	0.00437	0	NA
161	Glucan endo-1,6-β-glucosidase	*Haloplasma contractile* SSD-17B	7.54e-90	60.0%	EGM33352.1	0	0.00420	0	GH30
1785	Glucan endo-1,6-β-glucosidase	*Paenibacillus* sp. JDR-2	9.89e-34	61.3%	ACS99156.1	0	0.00895	0	GH30
319	Midgut trypsin	*Glossina morsitans*	9.35e-50	57.4%	ADD18692.1	0	0.00649	0	NA
321	Actin 5C isoform B	*Drosophila melanogaster*	0	100.0%	AAN09154.1	0	0	0.00403	NA

## Discussion

When compared to Zygentoma, even species in taxonomic groups traditionally considered as insect models for research on cellulolytic systems, such as Coleoptera, Blattodea: Cryptocercidae (formerly Blattaria) and Blattodea: Rhinotermitidae (formerly Isoptera), displayed significantly (>4-fold) lower cellulase activity. Species in Zygentoma are known to feed on highly lignocellulosic substrates [8] and to produce cellulase activity endogenously [12–14]. Recently, *T*. *domestica* has been identified to endogenous production of lytic polysaccharide monooxygenases (LPMOs) in addition to carbohydrate degrading enzymes [15], which may explain the comparatively high cellulase activity in this group.

Both *T*. *domestica* and *C*. *longicaudata* had similar digestive system morphologies, and the highest levels of enzymatic activity against the tested substrates were localized to the foregut. However, we detected significant differences in PCWDE activity between these species, with *T*. *domestica* displaying the highest and most diverse activity. Interestingly, *T*. *domestica* had significantly higher endoglucanase, β-xylosidase, and polygalacturonase activity levels.

Localization of highest levels of PCWDE activity to the foregut in both *T*. *domestica* and *C*. *longicaudata* is in agreement with previous reports documenting higher endoglucanase and β-glucosidase activities in the foregut compared to other gut tissues in *T*. *domestica* [12] and *Acrotelsa collaris* [20]. In addition, cellulose fibers were reported to be digested in the crop of *C*. *longicaudata* [21]. Similar localization of PCWDE activity, specifically endoglucanase (CMCase), to the foregut has also been reported in other insects such as desert locust (*Schistocerca gregaria*) and a longhorn beetle (*Hylotrupes bajulus*), as well as arthropod groups such as millipedes (*Chicobolus* sp.) [22]. In contrast, Lasker and Giese (1956) reported cellulase activity in the fluids of *C*. *lineata* was localized to the midgut and not the crop. Recently, expression of LPMOs in *T*. *domestica* was localized to salivary glands, and crop, with relatively higher expression in midgut tissues [15]. Interestingly, in our histological sections the columnar cells expected to secrete these enzymes were only found in the midgut epithelium of the inspected species. Although it is possible that in some insects the enzymes may be produced in the midgut and foregut (and crop), an alternative explanation considering the histology observations is that enzymes may be secreted from midgut cells but flow towards the foregut [23,24].

Diverse PCWDE activities, including cellulases such as endoglucanases and β-glucosidases; and hemicellulases like β-xylosidases, were found in the digestive fluids of both *T*. *domestica* and *C*. *longicaudata*. While this indicates that both *T*. *domestica* and *C*. *longicaudata* have all the necessary PCWDEs to digest complex cellulolytic substrates, *T*. *domestica* also displayed polygalacturonase activity. Comparatively, *T*. *domestica* had significantly higher levels of endoglucanase, β-xylosidase and polygacturonase activities, which suggests a more efficient cellulolytic system compared to *C*. *longicaudata*. In both the insect species, endoglucanases were found in the fluids of both foregut and head while β-glucosidases were found only from the fluids of foregut. This indicates the sequential localization of different types of cellulases in the digestive system of Zygentoma. Endoglucanases are the first enzymes to attack cellulose chain and break it down into smaller fragments recognized by exoglucanases (which were not examined in this work), while β-glucosidases are required for the final step of cellulose degradation to release glucose.

Feeding *T*. *domestica* and *C*. *longicaudata* on a high cellulose diet did not result in increased cellulase activity, which suggests that cellulase production in these insects may not be driven by diet. Similar results were reported from a gut proteome analysis of *T*. *domestica* fed on different cellulosic substrates, which did not alter production of carbohydrate digesting enzymes but increased abundance of LPMOs when fed on crystalline cellulose [15]. Consequently, it is plausible that in Zygentoma the production of cellulases remains constant irrespective of the diet but that the production of LPMOs could be driven by the content of lignocellulose in the diet.

Identification of proteins in chromatographic fractions with CMCase activity from gut fluids of *T*. *domestica* revealed the presence of endoglucanases with similarity to enzymes from termites, beetles, and the herbivorous crustacean *Daphnia pulex*. Although poly RNA was selected from the total RNA when preparing the *T*. *domestica* gut transcriptome, we detected two glucan endo-1,6-β-glucosidases matching to bacterial genes. One plausible explanation for this finding is that these bacterial glycosidase genes were horizontally acquired by *T*. *domestica* from symbionts, as previously suggested for stick insects [25], beetles [26, 27], and collembolans [28]. This explanation suggests the importance of bacterial enzymes for digestion in firebrats. However, previous reports suggest that treatment with antibiotics reduced gut microbial load but did not alter cellulase activity in gut fluids [14], which would support higher relevance of endogenous cellulases in the insect.

Overall, our work supports our contention that the digestive fluids in members of Zygentoma contain a repertoire of PCWDEs, including cellulases, xylanases and pectinases. Digestive fluids of *T*. *domestica* appeared significantly more active than in *C*. *longicaudata*, although in both insects the highest levels of digestion were detected in the foregut. Considering the results in this work and the dearth of information on Zygentoma, we recommend further research in this group that can be expected to reveal more insights into the evolution of PCWDEs in insects.

## Supporting information

S1 Table Sequences of contigs matching to proteins identified by nanoLC/MS/MS from gel bands within the CMCase activity area in zymograms of fractionated gut fluids from *Thermobia domestica*. (DOCX)
