# Supplementary material for: The digestive system in Zygentoma as an insect model for high cellulase activity
Source: PLoS One. 2019 Feb 28;14(2):e0212505. doi: 10.1371/journal.pone.0212505 (PMC6394914; doi:10.1371/journal.pone.0212505)
Supplement: S1 Table — (DOCX) [file pone.0212505.s001.docx]

Contig 23-endoglucanase

aaggtataaatacagtttcctgacaccagttaagttgtcctgtctcatcttcatcatgtggaagtcagctctcgttggtttgttcctcgttgcctctgccactgcgcagtacaactacgcagaggtattggccaaatctttgttgttctatgaggctgagaggtcaggaaaacttccatccgacaaccgtattccatggaggggagactctgctctcaacgacaagggaaacaacggtgaagatctcacaggaggatactacgatgctggtgaccatgtgaaattcaacttccctatggcttattccgtgactgttcttgcatggggttacctctccaacaaggctgcctacgatgctgcaggtcaaactcagtatttgaagaataccttgaagtggggcacagattacttcatcaagtgtcacgtgagtgccaatgaattgtacggacaggtaggcgatggaaacaccgaccacgcttactggggaagaccagaagatatgacaatgaacagacctgcttggaagattgatacttctcacccaggatctgatctcgctggagagaccgccgccgccatggcagccacagccgctgtgttccaagattctgatgccggttacgcctctaccctgatcaaccacgctaagcagctcttccagtttgctcttaactacagaggaaagtactccgactccatccagaacgctgctcaattctacagctcttctgaaatcaaggatgaaattgtatgggctgcagtttggctgtacaaggctaccaacgaccaatcttacttggaccaagccgagagcttgtacagttcattgggtctggacttccaaccatctgaattctcatggaacgacaagaccgttggagtacagctcctgttggccgagcttactggtaagagcaactacataagccgtgtcaccggattctgtgactacatgaagaacagtcagcagaaaactcccaagggattgctctacatcagtcaatggggaactctcagacatgctaacaacatcgcttggatctgcttggaggctgcaaacaaattgaacgtcaacactgacagctacagacaatttgccaagagccaggttgactacgctttgggatcttctggcagaagctacgttgttggatacggaaacaacccaccaactcatgaacaacacagagctgcttcttgccctgaccgcccagccacctgcgactggaatgtctacaacggtggccaacctaactaccaagtgttgaatggtgccatggttggaggccctgaccagaatgacaactaccaagacgtgaggagtgactacgtccacaacgaagtggcctgcgattacaacgctgccttcactggctctttggctgctctcagcgctctctactaagtgatagtatctttcttccaaagtaatatcatggcaatgacttcaggatccaataaacttacaacattttggaaccgaatttctaacactgaaaaaaagcaaatactaacttatgcattactttagtaaaaaaaaataaagttatgttgatttagtcaaaaaaaaaaaaaaaaaaaaaaaaaaaaaaaaaaaaaaaaa

Contig 309-endoglucanase

Aacaactagggaagtctttgttgttctatgaggctgagagatctggaaaacttccagccgacaaccgtattctacggagaggaaattccgctctcaacgacaaaggaaacaatggcgatctcatagagggatattacgatgctggtgatgatgataaattcaactaccgtttggcttacagtgtgactgttctcgcatggggttacctctccaacaaggtcggctacgatgcttcaagtcaaactccgtatttgaagaatacattgaaatggggaacagactatttcctcaagtgtcacgtgaatgtcaatgactcttacggacaggtggatgatggaaacactgacaatgctggtgatccagtaggttctgacctcgctggagagatagccgctgctttggcagccacagctgcggtgttccaagattctgatgccagctacgcgtccgtcctgataaaccatgctaaggaattgtttgagtttgctctcaacaacagaggaaaatactccgactctatccaaaatgctgctcaattctatagctctcccgaatacaaagatgaacttgtatgggctgcaatttggctgtayagagcawccaatgacc

Contig 62-α amylase

gtcaacttaaaaactaaaattactgacgcagctgctttatatgtctctatatttaaacgcgaaaaggtttccttccttctcatttgacctgtgtagatcaggcagtggcagtggtttcgtaggagaagatgaagtattttatagtcttatgtttgatgcctttgctgacaacgccacagaaaaatccaccagacaacaatacgaaaccgatcgttcatctgttcgaatggagatggcaggatgtggctgcggaatgtgaaagatttctggcaccacatggatttggaggtgtacaggtctctccaccgaatgaaaacataatagtggctaacagaccatggtatgagcgttatcaaccaatttcttacaatttaatcacaagatctggaaatgaagacgaatttaaagatatggtgaacagatgtaacgctgttggagtaagaatttatgccgatttggtgataaaccatatgagtggtgacaatcctaatcctgtaggaacaggtggaagtactgcagatccaagcaatagacaatacccagctgtcccgtatggaccaggtgattttcacagtcgctccgattgtccctcgtccaccggaggaatagaaaattacaatgatgctagtcaggtgagaaactgtgagctgtcaggccttcatgatctgaatcatagtaaagactgggtgcgacagaagattgtcgaatacatgaacaagctgatcagttatggagttgctggttttagagttgatgcgtccaaacatatgtggcctgctgatctggaagctatgtacagccaagtcaatgatttgtcaacggaacatggattcgcagctggtcaacgacctttcatctttcaagaagtgattgacttaggtggtgaggctgttcacggttccgactatacgaagctaggcagagtaactgaatttaaatatggctccgaattagggaatgccttccgaggacacaatccactgaaatggcttataaattttggtgaggaatggaacatgctgccatctggtgacgctttggtctttgttgataaccacgataatcagcgtggacatggtgctgggggctctaatatactcacatacaaggatgccaggctctataagatggctgtggcatttatgttggcgtggccatatggattccctcgagtgatgtcaagctttgcgtttgatgatcctaattccagtcctccagctgattccaatgccaacactcttcctgttattataaacgacgatatgacatgtggtaacggatgggtctgcgagcatcgatggagagagatcttcaacatggtcaaattcagaaacgttgttcaaggaactggagtgaacgatttctggtcgaatgacaactatcagattgccttctgtcgcgggggaagtggtttcattgcgatcaacgacgacagttatgatttgaagcaaactctacagacttgcctcccagctggaacttactgtgacgtcatcagtggagacaacgtgaatggccaatgtactggaaaatctgtagaagttggaaatgatggcaaagcatatattgagatccttacaagtgatttcgatggagtcttagccatacataccgagtccaaattataattcacaagctaagagaagcagaacaacattgaatgcaatttcaaacaggaaatgaattaacggctgccttgtggaaaagatacgtcaagaacttcttaatattatgtaatgtctatattacgtaataaacagaaccgtaatcttcataacttccttggtacaacaaaatctgaaattaaatgtaaattatctgtattttatcaataaaatattaatgaaaaaaaaaaaaa

Contig 56-endoglucanase

ttttttttgtgtttattatttatttatttatgcaaagccgaaaaatacattcttttgatgatgtggaacaaattaggcatacagaaatttgtagcaaaaatctacagtgagttttcttattgtaaaaatagactaaacaatggtttagactatcaggtttcaatcagtcttttaactttagtgagagctgaaataggccaagagaccagtgtatgctgcgttgtaatcacaggttggttcgttatgtgtagcgtccttcctgatgtcctgataattatcattagcatcaggaccaccagtcatagcaccagcaagtacgtgaccattgggttgattgccgttataccaattccagtcacaggcttgtggtgggttagggcatgatgctggtctgttgtgaacgtgagttggtggatttgttccccatcctacgatgaagcttcttccggtgtcacccagggcgtagtggagttgagattttgcgaagttgacgtattgagttggatttccaatattggccctggcagcttctacacagatgaatccaatgttgatagcccatcgcagggaaccccagtctctgatgaaaactaatcctttaggagtcctctgttgttgattcatcatgtagtcacagaaactctgaacttgagtcttgtattcggtctttccggtcaattgagccaaaagaacttgaacaccagcagccttgttgtcccaaccaaaatctggaggtcggtaacccaagtagaaactgctgtagaagttctcagctgcagttaggtagctctgttcatttgtagccttgtacaaccatagagctgcccagggcaattcatcagtgtaatcataggagttgtaccacaatgcggcatcagcaatagcatctgagtattttcctctgtacttatcagcaaaatcatacagttgtcttgcatgactcaacaaggttgcagagtaagaagcgtcgtattgcttgaagaccatggaggcagcagccatagctgcggctgtttcaccagctaagtcagaacctggatgttgtgcatcaatgtaccatgctggtctagcttctgtcatttcttcaggtcttccccaaaggctgtgatcaagagttccgttaccgacttgtccatagtacttagtagggctcacatgacacttgataaaatagtcagttgcccatttgatggtgttcaggaaatattgtacttgtcctgcatttacatatccttgttgattggacaagaagccccatgaaagaattgttacagtgtgggccataggaaatccaaacttcaccaagtcaccagcatcatagtatcctcctgtgagatcctctccattctgtccgcggtcattgagggcagaatctcctctccatgggatgcggttgtcggctgggagctttcctgatctctgcgcctcatagaagagcatagacaggccaatcagctcattgtagttgtattgtgcaactgcaatgtggcctgttacgaccaataacagacacaaaaccgttgacttccacatggtgaggagattctg

Contig 359-similar to luciferin-regenerating enzyme

catatggacaaatcatacaatattttccgaaaaaaatcgtttatgaagattgatccaatttcattacgttacaatattgtgtgattatacaaatatcttcatatatttcgccatttacaaaccttcacaatttttagaatagatcacaagtataattaattgatagtgctcagtgtagtttcatacaatcataatgaaatacttctttccaaacggattgcttatcttagtgtctagtagtattaatgcaagatattttatttatgttgtttctccctaaataagtactaaactgtttttttttaaaattaaatataatttaataatatatgaacagacgatagcaagaactgataacaaacatgtccaaaacaggcatttttcctgggaacttcgaactatattaatctaagacgtttcaagattggtcagtgttcggtcatttagcctactctctcgaatgtataacgacatatttctttttcttcttctttttttaattaagtgaaatcaacattttgtacttgtccaggatttgttgtaaagtattcaaatataagtttctcttatgcgtgattatattttggttttgtgttacgcctatttattatttttctatactctaagattttgacgtatcaatgtgctggccggaagtaactattttgaatgtagcgctgtctaaacattatagttgaatgtttgacctggatagcccttgacaccaagacctgtgacacggaaaacagctccagccttttccgagtaatgattcaagtgagctgtggtcacgtagagttcgtccaagttgggtcctccgaatgctacagatgttacttcttcggcaggaatgtcaattgttctcagcaattgtccggtccttccgtcaacgttcagcacttttgagtatccaaaggttgcgacccagagattgccttctgtatcaatagtcattccatcgggatcaatgccgtagataccattggcctgaagatcaaagaacgtccttcgattgctgatgtttcctgctttgatatcaaagtcaaagacgtcaaccctcttagttccgctgtcgatgtaatacataagagtgtcatctgcgttccatgcaagaccgttggaggtggacacttgagtcacctgtgttcttagaggttcattctgagcgatgctgtagaaagctccatcttgccagttgcccatggtgccagcccagaggcgtccacttcggtctgctttcccatcgttgaatctgttctgtggtttgttttgctcaacggttgcaatgacgttggtactggcagcactgttgtttcttccatcccaagtgagaattgcaagagtgcgaccaagtccaatcacgaacttattgctttgaccagtcacaggaacaaccaaagtcacaggaccatcgtttatgcgaatttgcgtgaattccttggtggaaggatcatatctgttgacagcctgtccggcgatgtccacgtagtagagcacttgggcctcggagtcccaatgaggcccttccgaatgagtcagagggctggtcacttgctccaccactacttgacaggaaacagaccatattcccacaaacaggaaagttatcacagcaataaccatttttatcatcctcgctagccaacctgtccttgctgcttcgtcaacgttaggatggtaaaaatggt

Contig 444-similar to luciferin-regenerating enzyme

Ttttcaaggaataaccagactcgtaaacctttattcaattaaccaatttacaaattttaatcaaaaactttcggaacaattaaacccaaagatgtttttatcttttattggattgtctattttatttgagggaaactcttggccggtagaccctttgctccaagaccagtcactttgaatgtggttccagcgagaggttcattcttcaaatcgatatctctggacgctgaagtaacgtaaagctcatcgaagttggcaccaccgaaagccactgacgttactttagacgcaggaatcttaacagtcgtcagcagagctccattgtttggatcaattcgtagaacctgtccaccgtcaaatgtggcgacccacaactttccttctgtatcaatggtcatcccatcaggaacgccaggtaagttgtgtttctgtaaatcgaaaacagttcgtcggttgctaatttttccattgttgaggtcgaaatcaaaagcctcgaccagcttcgttgggctatcgatgtaaaagaagatttgattgtccaagctccatgtgaggccattggagatgctaatctgtgacacgtgatgagtgagcttcttatcgcttgtaaaagagtacaggtttcctcgattattagggattcctgtcttcaaaatgggacccatagtgccgatccacaagcgtccactggcgtcaattttcccatcattgaagttgttgtctggtttttcgttctccaccgtcacgagcgcctccccaacttggaaaggtccgtcgtcttcaccattccacgtgagcacaaccaagtctctgtttctcggtatcaagaacttgtcctttgtgccttcgatcggaaacaccacagatgaataaaggtctgttttgatcttcgtatgtcttccggtcgctggctcatatcgatgcatcactctgcctggcacatctgggaagatcaaggcttgggcgatatggtcccagtgaggaccttctcccaaaacacagttcttcacaattggaatcacttgtggggaagacattttcaggcttgacttccgctgcactccacaactaaaacgtgagtgtgactaccgttgtacttcaatagagaaatcacgccactgcatagcaaccagcttctcaattctcatcraaaggtgactcgctgccaa

Contig 50-endoglucanase

tttttttttaaacttttacagtttatttgcaatgttttttgaacacacacacagacaatcaacaaatacattatagaaggttggtgttagaattatttgtacattgatcttgggtgttatttggtcacttaaatgagagctgcaagggcactggtgaaggcagcgttgtaatcacaggctacttcattgtgtgttgcatcttgtctagtatcagtgtaatcatcattggcacctggacctccaaccaaagcaccgtacaagatgtgagagttgggtctgtcactgttgaaaaagttccatccacattgttcaggaggtgaagggcatgaggctgctctgtgatgttcgtgtgttggtggattgcttccgtatccaacgacgtagctcctaccaccacttccaagagcatagtccacttgactcctcagtaattgtctgttagcatctggattgataccagcttcggcagcttgcaaacagatgtacgttgctccaatagcgtttctaattgatccccattcaccaaggaagatcaaacctttgggtgttctttgttgccgagtcctcaagtagtcacatgctcctgagacaagattcttgtaaacactcttgccggtcaattgagccaacagaacctgagcaccaaacatcttgtcatcccagccataagaagtaacttgataatttccaaactggttgaaatagttctcagctttagtcagataagtttggtctttcgtaagtttgtacaaccaagctgacgcccacacgagttcatcattgtaagaataagaggcataaaattgagcagctgccgctacagagtcggaatatttggctctgaaattttcagcgaattcatacagttgaatagctctttgtctcaagttgttggcgtagtttccatcaacgctttggaaaacaagggaagatgcagcaaaagcagcggccacttcaccagccatatcagttcctggatgtccttcatcgatcttgaacgctggtcgctgcatggtcatactttctggcctgtcccaagagctatggtcgacattaccatcaccaacctgtacatacaattcatgaggagctacatggcacttgatcaggtaatcaaggccccatctcaaagtgtcataaccatagttagtttgtcctgctgcatcatagccggacttgctggtaagcatgccccaggcaagagtagtgagcgtgaaggccatgggaaagttgaacttcacattatcaccagcatcataatatcctccagttaaatcttccccattgttacccctatcgttcaaagcagagtctcctctccacggaattctgttgcttgctggtagctttccggatctttgggcttcgtagaacagcaatgagtcagtgagagctaacttatagtcatattgggcaactgccaaatgactgcagaagaccagagcaatacaaacaaccacgaacctcttcatgatcatggaaggtggattctcactctcgagcaaacacttt

Contig 11-endoglucanase

cccgtcatcttcagtcaccatgaagtggtccgtggttgtttgcattgccctggtcttctgcagtcatttggcagctgctcaatatgactacagcttagctcttgaggacgctttgctgttctatgaagctcaaaggtcaggaaaactcccatccaacaacagaatcccatggagaggagattctgctttgggtgacaagggtatcaacggtgaagacctgactggaggatactacgatgctggtgaccacgtgaagttcaacttcccaatgtctttcaccctgacaactcttgcctggggtatgcttaccagcttacctggatatgatgctgcaggacaaactcaaaatggttatgacgcactgagatggggtcttgactacctgatcaagtgccacgtgtcagaacatgaactgtacggacaagttggtaacggtacattggatcataactactggggcagacccgaagatatgaccatggaaagaccagccttcaagatcgacgaagcacatccaggtactgatctcgctggtgaagttgccgctgcttttgccgctgcttctcttgttttccaagatgtcgatgctaactatgccagcgacttgagacaaagagccattcaactgtacgacttcgctgataacttcagagccaaatactccgactctattgaggatgctagtcacttttacggctcttattcttacaacgatgaattggtgtgggcatccgcttggttgtacaagcttactcaagatgaaagctacttggccaaagctgaggattacttcaaccaatttggaaactatcaagtcactggatttggttgggacgacaaaatgtttggtgctcaggttttgttggctcaattgaccggcaaggatgtttacaggaatcttgtctcaggatcatgtgactacttgaggactcagcaacaaagaacacccaagggcttgatcttccttggtgaatggggatcccttagaaacgcaattggagcaacgtacatctgtttccaagctggccaagctggtatcaacccagaggttaacttagcactgttgaagagccaaatcgattatgcacttggagatggtggtaggagctacgtcgttggatttggagagaactacccaacacacgagcatcaccgtggagcttcctgccccactccaccacaagaatgtggctggaatttcttcaacagtgatgccccagactcccacatcttgtacggagctttggttggaggtccaggtgccaatgatgactacattgataccagacaagatgctcaacataacgaagtagcctgcgactacaacgctgcctttaccagtggtctcgccgctcttgttgaaaataccaaataatataccaactcttgtaaaatgtagctgtagacttatgtacgtttcaaaattgtaaataaactgtattatttcaaaaaaaaaaaaa

Contig 9-endoglucanase

tttttttttttttttaaattcaaacctttaattttattttactttcgaattaaatttcaaggtgaatttcacatttctacatcattttcaattttttaatgtgctatttttgcattttagaggagtcctttcatttggagggtcttaataccagcaagagcactggtgaatccagcgttgtagtcgcaagcaacttcgttatggacgtagtcggttctgacatcagtataagcatcgttctcatctggaccacctaccaaagcaccatacagagtatggggatttggattagggctgttcaaagcaccgtcacaagatgctggtgggtcagggcaagatgctcctctgtgatgttcatgtgagggaggattcacaccatatccaacgacgaaactcctaccagtgcttcccaaagcataatcaatttgctgtttggcaaaggttctgtagtctgcggcgttgtcaattccagcgttagcggcctcaagacagatcagagcaatattggaagctacacgaagagttccccattggctgatgtaaatcattcctttaggagtcttctgttgttcgtacaccatgtgatcgcagaaatctttaatttggtttacatacacatccttgttggtttcttgtgccaataatacctgtgctcctgcaagtttgttgtcccaaccaaaatcataggctctgtaaccgaggttgaactgttggtacaaactctcggctgtgtccaaataggtttggtcattggtggccttgtataaccatacggcaccccacacaagctcgtcctggtagctaccggaaccatagaagccgccagcagcacccatagattgagagtaaattcctctgtagttgtaggcgaagtcatacaattgcttggcgtgatccaacagagttgcagagtaactggcgttcacgttctggaaggcgatagaagaagcggcaagagcagcagctgtttcaccggcgaggtcggatcctggagcattctcatcgatcttgtaggctggtctaggcatggtcatgtcttctggtcttccccagaaagcatggtcagcattgccatcaccaacttgaccatagaaggtggtaggagctacatgggccttaatgaaatagtcagttccccatttaagggcagccaaaccatattccaattgacctgcatcttcataggctgcttggttgttgataataccccaggcaagaacagtcactgtatatgccattggaaaaccaaacttcacgtgatcaccagcatcgaaatatcctccagtgaggtcttctccattctctcccttgtcaccaagagcagcatctcctctccaaggaatcctgttgtctggagggagttttcctgatctctcagcttcatagaacagataagataaacctaacacctcgccatagtcgtattgcgcagctgcaaaacgacctgaagccaggaggaataaacagagcagtgttggtttccacatgatgatccaagacaaccctctccctt

Contig 253-endoglucanase

Cgggttgtttccgtatccaacaacgtagcttcttccagtagatcccaaagcatagtcaacctggctcttggcgaattgcctgtagctgtctgtattcacatttagtttgttcgcagcctccaaacagatgaaagcgatgttgttagcatgtctgagagtaccccabccg

Contig 5-chymotrypsin

Cccacggccaattccctgcgcaacagattactccagcatgaaggttcttctcatcgtcactattgctcttgcagtggctcaggccctgccacagaaaactctcattccaatccgaaagacccttccagtcttggaaacggctcctttgccaaaattgaataatagaattatcaatggcaacgttgcctcccacggccaattcccttaccaagttgccttgatcatcaatggaaatactttctgtggtggatccctcatctcaaataggatcattctcacagctgctcattgcgctgagggagcttcatccatcgaagtatacttgggagcccaaaacatcaactctccaagtgagtctggaagacaaatcatcgctgccgatgccgctgcagttcacgaacaatacaaccctaacaccatcaataatgacatcgctgtcgtagaactctctcgggccgtcagcccttccacctacattcagcccattgatcttgaaagctcggttagcaatctcgaaggtgctaccgtcaccgtcagtggatggggaaagacttctgacagtgcaagtggaatcagctctgaactgagatacgttcaattgccaatcatcagcaactctgaatgttcacaatactacggaagctccatcaacagcggaaagatctgcgctgacggcaactacggcagctcctgcaatggtgacagtggtggccccatggtgcgtggaggcactcaagttggaattgtctccttcggaagcagcagaggatgcgccagtggatacccatctgtttacaccagggttggctactacagaaactggatctcccagaccgctggagtctaattggcttctattcttactttacataaattgtaaaataataatgaaaaataaacaacatatcaagtaaaaaaaaaaaaa

Contig 4-endo-β-1,4-mannanase

cttgattagtacatttcgtcctaaacatgaaggcttgtcttgcaggcttgtgctgtgtggcacttctggcatcactgttgccaaaggtaaatggatacctgaaagtcagcggtgacagtttgacctataatggacaaaagatattcctgtctggacccaatattgcctggaattgctacggatgtgactttggcaatggtcaatatgataaaagtggtccaaacttagaagattggttgaaacagattggtaatgccggaggaaatgttgcacgtatctgggttcatattggcgcccaaacaactccagccttcgatagcaacggatatgtatctgctccagacgccggaggtactcttatcaacgatttgaaaagatatttagatgttgctgctcaacataacattattgtcatctttgtactatggaatggagctgctacgcttacaccagagcttataggactcattacggacgacaacaaactacagagctacatcgataatgctttgacgcccatagtgaaagctttgacctaccaccctgcactgggagcatgggaaataatcaacgaaccagctggatcatggggaatcacatataactctaatccttgtgctgatagttctccactggaagctgcaggagctggctgggccggaaatgttctaccagaaagatctttgaaattcatcaattggcaagctgatgcaatccatcaaatagattccaacgctgctgttacagtgggaacctggtcatcaaatccaatttctgacaaaattggaaagaggaactacttcagtgatcactgtctgacgagctttggaggtagaagcaagggaactctggacttctatcaggtgcacacttatgactggcaaggtcaactgcctagcaactctcctttcttggtgaaatcttgggactttggccttgataagccattgatcattggagaatttgccagcgtttgtgctgcaggacgtaatatcaatgaacttatggactacgcgtataacaatgggtaccaaggagctttgacttggcagtttaatgctggtggagaatgttctgattcacaagctacccagttgcaagccttccaacacatccgtagctacagtggcgtacctatcagtgtctcaaaataaacccaagagggaaataaaatcctagtacagatatttcaaattatgaacagtacaatttgcaaccttaccagaactgaataaaactttcatgtgatgaattataaaaaaaaaaaaaaa

Contig 25- endo-β-1,4-mannanase

gtcatgaatgatgagtataaaaacggttggttgaccacacgcaatactttttgtcccaatcatgaaggctggtattataagtttatgttctgtggcgctgctagctattgtagtgccaagtgcaaatggctatttacaagttagcggtaacaacttgacttacaaaggacaaagagtatttctgtcaggatcgaacattgcctggaattgttacgcttgtgatttcggtaacaaccaatatgctcaaaatggcccaactcttgaaaattggctacaacaaattggcaacgccggaggaaatgctgtccgtatttgggttcacattggtggagaaacgacaccagccttcgacagcaatggcaatgttgttggtccagacaccggtggcactttgattgacgatatgaggaagtacttggacgctgctgcagcaaatgacattcttgtgatctttgtcttgtggaatggagctgttgcacctcaacaaagagtcttgggtctcatcaacgacgacactaaattgcaaacctacctggacaacgcattgacgcctatggtacaggctttgagtgaccatcccgctttgggagcatgggagattatcaatgagcccgctggttcctgggccatacaatctaattctaaccaatgcgctgacacttctggtctacaaggaactggagctggatgggcaggcagtatctctgctgaaaggtctttgaggttcataaactggcaagcggatgcaatccatcaggcagcttccaatgcagccgtgacagtcggaacctggtcttcaaatccaatctctgacaacattggaacaaggaactacttcagtgacgaatgtctgactagtgttggaggtcgaagcagaggaacattagacttctaccaggtccatacctatgattggcaaggtgcttttgctggtaatgctgcttttgtggtgcaagcttctgatttccaactggataaacccttgatcattggagagtttgccagcatatgctctgcaggacgcaacatcaacgacctcatggattacgcctataataatggatatcagggggccctaacatggcaatataatgcaggaggaaattgtgctgattcccagaacactcagcagcaaggcttccaacacatcagtggctacgatggtgttagcatagacgtctaaaaatactcctttattgactgaagtacacactgttatgcaattcacaaaaatcttgtattaaaatgtcaattatataataaatttcataccatcaattattaaaaaaaaa

Contig 488- endo-β-1,4-mannanase

ttgtaactcccgcagaggattgtatacaaatacgctactcggaaagctttcttgttcaattcagtatgaaagtcacacaagtaattttgatttgcagtttgctatatgtgctgtcgacagtggcagaagcttatttagaagtaaacaatggagttctaactttccgtgggcaaaaggtatttttatctggggcaaacattgcttggaaatgttacggatgtgactttggtaatggacaatatgcacatagcggtcctgtccttgaagactggttaagaaaaataggaaatgctggtggaaactttgttcgagtatggttgcatattggcggagagacaactcctggctttgatagtaatggctatgtcactggtccagactgggatggcacgattatagatgatatgaaaagatttctagatgtggctgctgaaaatgatatccttgttatgtttgtactgtttaatggtgcagtggctcctctatcatgtgctcagggtctcatctatgatgacaataaactccaaagttatctggataaagcactaaagccaatggttaaagcactatctggtcacccagctttaggtgcctgggaaatagtcaatgagcctggaggctcatggaatgttgagtacaacactaacccttgtgcagattccacttacctctcggacactaaagtgggttggtctggagccaatattacaccatggaagtttttgagatttatcaactgggtagtggacggaattcatctagctgacccaaaagcgatggtcactgttggagcctgggacacctatcctgtatctgacgccataggcaaaaagaattacttcagtgatcactgtttgatcagtgttggtggcagaagcctaggaaaactggatttttatacagttcacacttatcctgattacagtggtcattttccaagcgtcggggcatttaatgtgaaagcttctgactttgggcttgataaacccttagtaattggagaatttgccagcgtctgtgccgaagggaggaaggttaatgacctattggattatgcttattacaacggttaccagggagcactgatttggcaatttaatgaatctgatgactgctcagatcctcagactgttcaagagtcgggatttcaacacgtaagatactacaatgatacttcaaatggaggacttgtagccatagacgtctagttgaaaagaagtattgtgattgtatt

Contig 61- endo-1,3(4)-β-glucanase

tttttttttttttttagatttttttcctttgtacgatttgcaggaactatttattaacagtacaacaacacaattggaatgaaattacagtctatttcaatattttatttaattaaagttctaaatttattttgtgcattccaatgtttatttctactttaaattggaaaatatacttcgctgactgcaagaaaaaacaatttacaacacgagatgatcataaaagaaataacttatggtagtgaccaaactcggacataatcgatctgcaaggcggcatcttcatcatgccatgtcgactgccagcaacttctgccttcccagaagtctctctttggatgtggtgagcctcctgaccatggcttaccacatggattgtttggaacgccatctgggaagaaactacctcctacagctacgttcaagatgaagtagaattccttatcaaaaggtgctactaaagctcctccagcccatgggtttggaccgcctgggtcgttttcgaagcctcctaattgccagaatccacctgcaggtggagtgactgatccaatctccgctccatcaaaatagaaggtaatagtatttgggttccaagaaaagccataagtgtgaaaatcagctgcgaggtctgtgccagtaacttgtttttcccaatgagttctaggatatccgttgtatggccaacgtggaccccagtgaagagtggaaccgtaccactgaacaccaagatttccgtagttttggtttccacgactttccattatgtctatctctccagatgctggccatccgccgtagacgctgtccctaggaagcatccagatagctggccacaaccaatctcctctaggaaccttagctcgtatttcagcctttccatatgtgaagctaaatgagttgacagtcatcagttttgctgattgcactgggttgataatttcatctcctccagttgcaatacattctgaactgcacccttggtttccaagatttagagttccgtgatacaggaaatcttctccaaattcatcggctgtaaacgtcggtttgatgtaaagaattccatctctcacgtaactgttgcgtctgtcgttcctatagtattggaattctccatttccattaccggaaacagtcacagcatgcgtccatcttgattcgtccagagtatcaaaattctcttcaaatattacttgttgggctgacgctgacacaatcgtcagcaccaaacatagtgcttcgaggtaagccatcttctgtgactaactggctggtttatccagtcagtctaatctgaaatacgacgaaacgacacaagatctctt

Contig 65-acidic mammalian chitinase

atagtgctccgawcatcattcgtttatatctgtgatactgaacacttcttcagataacaatatgatgttcggagcactattaatcgcaagtttattttttgcaattcctccttctttgggtgcggaaaatcgagtggtttgttactttgctagttgggctacgtatcgtcaaggagaaggcaaatttgatgtagagaacattaacgtggatctatgcactcacatcatttacacttttgctggacttgattctacgactaatacaatcaaatccttagacccttggaacgatttatatgacaattatggaaaaggtgcatacacaagattcgtgaacttgaagaacaaaaatcccaatttgaaggctcttcttgcgataggaggatggaatgaaggatctgttacctactcagacatggctagaaacagtgaacatagaacaacatttgtggagagtgtagtagcatttcttaaagaatatggattcgatggtttggactttgattgggaatatccagcacaaagaggaggaagacccgaagataaggaacattacgctgccttactaggagaattaagagcagcattcaatgaggaaggccttatgttgacagcagctgttcctagtctgaagagttacattgatgcaggatatgatattcaagctttagttgataacctggatttcttgaatgtgatgtcttatgattatcatggttcttgggatggttataccgcactgaattctcctttatatgtaaaaccagagaatcagcttaatgtgaatttcactctcagctactatgaggaacaaggtgttcccgctgacatgttggttgtgggtattcctgcttatggtcacacttatacactcactaatcccgacaataatgaaattggcgcagctgcttcaggagctggaaatgccggaccttatactcaggagccaggaacccttgggttcaacgagattgagcaatcaatggttgataatccagatgcctggactgtagaacgagatgaaacaagtggatctccatatgcgtatcgaggagatgaatgggtgtcatacgaagatcctgtttcagcagaacaaagggctcaatatagcgtggaaagaggatatggcggagttgtagtctggagtattgaaacagatgatttcgttggatatagaaatgaaacattccctttgcttcgtgcaatcaacagagtgttcgatcaacagaagtaataacttcattaggcgttttgacctttttatttattgtaaaatattatttacttacttagttatctttgaccgtaactacttgcaataaaaatatatttaaatggcaaaaaaaaaaaaaa

Contig 234-endoglucanase

gtacaaggctacagaggaaagtactccgactccatccagaacgctgctcaattctacagctcttcagaatacaaggatgaacttgtatgggctgcaatttggctgtacagagcaaccaatgaccaatcttacttggaccaagccgagagtttgtacaatgaattgggtctcgactacaggccatctgaattctcatggaacgacaagacagttggagtacagttacttttggctgaacttactggtggaagcaactacaagacccgagtcacggacttttgtgactacatgaagaacagtcagcagaaaactcccamggggtcgcgg

Contig 1026-similar to β-galactosidase

attgcaatgagttattagaatctttgagcatgttgctgcctttgtactgccttggtgtttacctgttactgccattcgtggcagcacaggatttgcctactctctacgaatattacacggaaggtggaatcaaaagtggtattaacgcaaccgctgagggatttaaacttaatggaaaggatataactatctacagtggtgccatccattattttcgagtccaccctgactattggagaagcagtctcaggaaagccagagcagctggactcgtagccgttgaaacatatatcccttggaatctccatgaacctcgaatggatgagtacgatttcggagatggtgacaacgatatgtcagcgtttctaaacgtaacaagatatattcaaattgctcaagaagaagatctgctcgttattgtaaggcctggaccatatatttgctcggaatatgagtttggaggatttccaagctggcttctgagatataaaggaattggtacaagaacaaacgatcaacaatacgttgaaagaatgaagatatattttaataaacttcttcccttgttggcacctttgcagttcactgaaggtggaccaattatcatgttccaaattgaaaacgaatatggttacttcaatcctatttcaaaagactacctggaagccatccgacaaacctttattgataacggacttaaagaacaatttgtcacctctgatggaccatggcaaggagatgtgggaagccttcctggagtgttgaaaactattaattttaatagtgatcccaactctgccatacctcaattacaacaacttcaaccggataaaccattaatggcaatggaattttggtccggatggtttgatcattggcttgaaggacgcaatacaggacttaatgtcgacacatacgctacttactacgaaaggattctgcggttcaacgcctctgtaaatatttatatgttccaaggaggaaccaattttggatttctcaatggagcgagtctttcaggcaatactccagattattcatcaatagttagcagctatgattatgatgctcccttaagcgaagcaggagactacacagaaaaatatcagaggacaaaggatctaatcgcaagatataacaaaatccagacacggctcccagaccctccagaggaaacaccaaaatctgcttatccaacaacagctattcaggaatacttgtcgtattcggacttcatagatagagtaaatgaagaagatagagttcaaatagaagatgtagtatcgatggaagacctcgatgtgaatggtggtagtggacaagacttcggttatgttgtgtacagaaagaaccttcaactaaatgaagattctgtgctcaggatttcaggccgtgtccgtgatatcgccatacttcttgtggacggcgaaaggaggacgaaacaattctcaagtcgtgatgatatcaatggattcggttactgggccaatagcgatatggaatttaactttgatccaacagatgctggagatcgggttgtggatatcctggttgaaaattggggaagagacaacggaggcaatacactctggcttcgaccagccaaaggattatgggaaggtcctgtactattggatggagaaaatctaacaaactgggaagcaatttcgcttccatttaaatccaagtgggtcagaggattaactggctggagagaaattgatgagacagtcattggtccagctttattaagagcgaccttcaacgtcgattctcctgcagatacatttattgacatgagccaatggggaaaaggcattgtatttgtcaatggttttaacattggacgatactttaaccttgggccatccaaaacattatatgttcccgctccacttcttaatgaaggagaaaatgaagttattgtatttgaacactacgcaccagcatcagaaatagttttctctgctacacctaatttgggatgaggacaagatttcggcaataggtatccagaagacagacaaaccactagcaaatacattgctatttttatattgttagcgcr

Contig 68-chitinase

cggaaagaacagtcaagacagtttcaacttctggcacacttaaagcagaaacggacatccataagcatgagaaacattctacttataacgtatattataacaacaggaatattaagccaattagaggcagctaatatacaatcagatttgccacaacctggagatacacttgcgcgacgggcctgttattatcactcatggccggcttacaattccactccgtacggaatagaagatatcccaaccgatctgtgcaactatctcatgtattcagatgtcactgtagacaataagacatgggaagttctcatttataacccagagactgaacttgaccacgatggacagcccggagattttaggcgattcacaaatctaaagcaaaatcaaacaggccttaaaacaacgctttcacttggaggatggacttttgacgacggaaggaggttctcagaccttgtttcggtccaagagagaagagctattttgattaacagtattatcaatcttttggaagaatacaacttcgatggtctgaatttagactgggaatatcctggggatgcaactatgggaggaagaccagaagacaaggataacttccttttattccttcaggagctgagagcagcttttgacgaacaaggaacagggttagaaataacagtagtggttccagtcaatagaacaggcattgatgatggatttcatgtccaggaactctgcagtcttgtagacgcagtgtattgctacagctatcaacttcgaggagcgtgggatggatttgcagatgttcccagtcctctgcacgcaagacctggattcgaccaaggacattacatcaattatactgtggaaggaggtctattattatgggaagaactaggttgtccagctgagaagcttgcagtgggagttgcattctttggtcacttattcattctagaagatccagagcaacatgatttgggtgctccaattactggagagaataggcagaaaataccgtacttagagatttgtaatacaactttttctcaagttggctggcacagagagtgggatagtgagggtcttgttccttatgcttatagagatgaggaatggataggctacgaagatcctgtaagtataaggttcaaaatgaattttattaaagagaaaggctatgccggcgcaatggtatgggcagttaatttggatgattatttgaatagctgcggaggaggaattaatccgatgctgtctgttgtgcaggaggaattaggaaactatactgtgcctagaagaatccatcattgaatgttccgaagtaaatgggtctgccacgatactattccttactgttcaagttgtctaaaattgtctaaagaaatcgactttgattgttagtttacctatttgtatatacttcaataaaatattccgaaacaagccaaaaaaaa

Contig 54-chitinase

tgtcgtaattggtttcattgatttaaattttcacagaatcacaggccagaatcatgaggactgatcacttcctaacagtgttagcaggtcttagcctgtacttgaccgtatgtatggtctcgtctcagaccgctcgccgagcctgttactatgagtcgtggagaatctacaaccaaaccaatgaaaccaattacgacatccagaacattccaactcagatgtgcagtcatttaatgtatgccatggttggagtcaacaacacatcatgggaagttagagtcaacaatccagactacgaccttgatggtcgccttcctggcggtttccgaaggttcacagatctgaagaaagaggagcctggcctgaagaccactttgtcccttggtggatggtgggaagaagatgcagccaactactctgatatggtttctctcccagaaagaagggattcctttatcagcagccttgtagaacttgttctggagtacaatttcgatggagtggacatagactgggagtacccaggagatcctctacaaggcgggaaaccagaagacaaagagaatttccgtgatttccttcatgagctcagagcagctttcgatgacagcggaagggaaggcttagaaataagcgttgtagtacctattaatgaaacacgtgttagtgctggatacgatgttgaagatctttgttgcagtattgcagacgcattgtacactttcacttacgccttgcgaggacctgattatggatacgctgatgttccagctcccctccacccaaggccaggcttagacaacggaacatttgctaatttcactgtggaaggtggtctagcattatgggaaagccttggctgcccaaaagagaagctaatggttggaattgatgttaatggatacagagtccaattggctgatgagaacaaccatggattgggtgctccgatagtggatggacaagtgtacagcgaaataccttacgaagagatttgtacgacccttgtagttgaagatgggtggactagggagtgggatgatgttggaaaagctccctacatgtaccgaggtgatatttggataggatacgatgatcccgagagcgtaggttacaaagtagactttatcaaagagaatggctacggtggcgccatggtgtgggcagtgaatcaagatgatttcttggataattgtggcgatggagaaaatcctattcttactgcggtacacgaaggattgaggaattatactgtacctggcaatttaaagaggaagtgaagctcccacaatgaacttgatgtcagcaggcattcttcagctaaataccttccctcactgttgaaagatcttgccaatgagtagttggttgtaaaaatgtaaagaacaaatcgtgtgtgttataataaattctcgacatataaaaaaaaaaaa

Contig 157-hypothetical protein SINV 01527

tggagataaatattgcaaaatcaattaagatagattaaataattgtacatgaaataaagcttgcccatttcctacgtttttcatcgttgttgttcatcagccggtttctcatttgcgactgcaaccaggaactgcatcaggccaggtgcagactttgcccacaggatcaaagacggttcctgctccacagtgataggcaattgtcacccattctcctccttggatggaacattcatagtacatgttgcagttcgtaggatctgcgttcaagccaacttccttgcagatctcactgggggcgggggtaggagctggggaggttgttgtagttggggtagttggactcgtactgcgtggggttgttgatgtagtgctttgcgaagcatcagttgtagtagtaggtggtggaggttggtcaccaacgacaattcggttcaaagtgtgcaacaatgggttttcttcgtctccgcaattggcatggaagtcatctgtttctagtgaccataccatggctccagccaggttcatttcgttcacatagttagccttcacagtgatagattctacgtcatcgtaaccaacccattgatctcctttgaccatgtaaggtacttgataatatggatccctaacaacattccaaccaccttgttgcaacataacacagatttcattgtatcccaaaaatccagcttctctggtgtatggtccagctgtacatggctgagatccaggaactccaattccattattttcaggtttgtccaacttgaagcaccttccgtacaatgggattcccatgttgattttttcgggtggagctcccaaactgatccaatagtgcacactgaagtttgtgttgaagtagacgttgtcaccctgatcatcaggtcggccgtacatgggagagttaagtcctgttactttatcaaaggctccgtggtaatcgtagctcataaggttgatgtaatccaaagactctgccatcttgggtacatcgtaggcgacatcaattgttccttttccggcagatacagctgctgtcagcaagagtccttctggagcaaaagctgccttcatctccttcaaaagggcagcgtggttctctctgtcctctggtttgccacctcgttgagcagggtattcccaatcaagatccagtccatcaaagttgtacttcttcaagaatgtaatggcgttagtgatgaacttttctcgacttgcggcagtagctgacatgtcagaatattttgaagatccctcattccatccaccaatagccaggagtgttttcagacttggatttttctccttgaggccagtgaatcttacaaatgcacctttgccataattatctggtaggtcattgtagccatcaagtgcagtcatgattccatcccagctgagaccagcaaaaccatatatgatgtgggtgcacaggaaaggatcgatattttctacgtcatattttccaccttctggtcgatacactgcccagcttccataataacatactaaatttttaccgttagcaaaatgcacagcaaatgctgccaggaatgttacaagaccaaacacagcgaaacacttcatattgtccacttccttcccagtgagagagggaatacactgattactggtaaatcacgttcgcttttttatagtccttcaaaataatagcagccccgctccttcgttcacacgatgtggaagatgtgtgtgaagaaacattcattcgttgtttagaagacaatactgcacacacacacacacatacatacatacac

Contig 240-endoglucanase

Tacgccctgggygtagtggagttgagattttgcgaagttgacgtattgagttggtggatttgttccccatcctacgatgaagcttcttccggtgtcacccagagcataatggagctgcttccttgcaaagttaatgtattgagttggatttccaatattggcctttgcagcttctacacagatgaatccaatgttgatagcccatcgcagggaagctgcca

Contig 383-chitin deacetylase 9

aattattacgctcacaactgcacgggtattaaactattttctcaattttctcacagtcgtacattattatgtcgaataaaaataagaccaacataaataaaatattactgttaatatttttatatgaatgcagtatttattatcaaacaataaatatataacattaagaaattcaaattcagtacttcttacgggcaagtttcatgatgaagttgttgtcgtggctggtaatccttgtgggttaccgagccatggatatgcagcagggcagtgctcggaacaagtttgcatgtaaatttctgatactcctgccggagtatctgtatacctgcatgattgtcctcttccgcactcatcaggtggaagctcactctctggagtgcattgccatggggcgaaggagtcgatatctgacagcggtgttggatttttcacccactctaacatttgttgcacagtgattacatatacatcatcaagggttcccaaatattgaagaaaattttggtaagcagtccagtgataaggatagccaagtaaccaagctgcatgcatgaaaactccaaatggtgctcttgtctcactgtcgtagtgattatggaagttatccttcatctcctgaagtacaccctcggctgtatcattcataatacaggcatcaagcattgaacaaggagcgtcattgctgcccatccaatccaccataggcacaacccatgttcctgggtaggattctactggacattttccaaccggacagtcctggctagattggtagtccaaagtgtacggccatatgggtggagatctgtaagagtaagtaccccatgaacagtcgtattcgaagccgttatcatggagaactttgaagtaagtatctccgttcatgttaagaaacggaacccgaattcctttcatatcttccagggggataaggccatataaattaaacatctctctgtcacctacaaattcatcagatattacgtctgcggaagcgtctccttgttgggcatgcgttatagaatgaatggcaatttcatttcctcttctgtataagttataagcttttgtataatccgtgtattcgtgcgttgcaaaatatgtaacagtgactggacagccatttggattagtgtagttagaagcaatagggttgtagtaaaggtcgtatagctgtgctgtaattgcatcatcgaaactcagcattacaatctgaggaacttgttcaggagctaaatcaccaggcactccactgttgaaacatttacaatctggcaatttgcaagcattgttatcacattcttttgctgctcttggattaggcacagctgaaactgcagccacgataactaaaagcaaccccaaatacatcttgatttgtgagaataacaagtatttctggttctattattacgtgggagtatatatagttcaactatctgtcaa

Contig 161-glucan endo-1,6-β-glucosidase

accgtcgcattggacactaacacatcatgaagttattgctcctttctctgttagcagtttccgcttatgcagcatgccctgaggggcaattgcagtattggtcaacaaaccctgcagatctgtttgatgatgtaaacgctggtcagatccagggatgcttatctggagaagcattgaaaggccaagctcctcgtgcaggaacggttgaagtagatccaagtcaacgtttccaaacaatggatggcttcggaggtggactcaccaactcagctgcgtatctaattcactacagcaaactaagggacgaattattgcagaaacttttcagcccaacagatggcattggcatcagttatgttcgtcttccaatgggagcctctgattttatggcaagaggccctgcttatacttatgatgacgaatatgatccagacctaagccattttagcattgaatcagagaaggagtttgttataccatcgattttgggagcccgtgaagttaacccagatctaaaaatcatagccgctccatggacagctcctccatggatgaaagacaacaatgattacaacagtggccgttttctcactgaatacacagacacgtatgcagatttcttcgttaagtttctgcaaggatttgaggagaatggagcccatatagattccttgtctctccagaatgagcctttgctagaaacgccttacccttcaatgattctagaagctgacgctgccattaatcttattaaaaatcttggaccaaaacttagagataataacatcgatgtcaacttagtaacatgggactgggattttggtaacactgagtatgcatttcaagtactcaacgatagtgaagctagtcagtatgttgaaggatttgcttaccatggctatactggtgatccaaccaacccaaaaattgttcaggatgctttccctgacaaaagatactatttcaccgagttcactggaacaacagccgacaccaactggcccagcgtgtttagctacatgcttggaacattatttgtccaacaagtacaagagggagccgtcgctggaattctctggaatttggtcctgaacgaattctctggaccaacttacaatggagctggaggatggaaaaactgccgaggaatcctgactttatactccgatgatagccactatgatgtcagtgttgaataccacgcaattggtcacatgaacaaggctgctagccctggtgctgttcgtattggaagtccagaaaggtttggagaaactaacagccttggttccgtagcttttgtaaatcctgacaattctacaggtatccttgtgttcaactataacgctgaccaagctgaagatctggaagttagattgaatggtcaagtatataatttcggacaagttccaccaaatggtgctgtcactttcagaacgtaaagtatcacgcttaaattgtatttcgacagattattgtaaataaatatttttaatacaaaaaaaaa

Contig 1785- glucan endo-1,6-β-glucosidase

Tgcatgtgtagctgttttgtaatcaaggaaaaggaaaagtgaagttataacgtcaatataaatttgtcgtttacacgtgcataccactacagctggccacacaaagtataatgagggtaatactattgcttacgctggttgacatcgttctcagcgcttgtcctgctgggcaagtagaatattgggtgacagatccaaatgaccattacacagatgacacggcaggccaaaggcatgggtgcctcactccgagcagaagctccaatgtggaaccacgggcagctacaattgaaattgacagcaatcaacgattccaaacaatggatggctttggtgcagccatgtccaattctgcagcattcctcatacatttcagtagacatcgaaatgaacttctccagacattgtttggaagcgatggaataggcattaactacattcgtctcacaatgggtgcttctgactttatgggcagaggatatgcttatacttacgctgatgaatatgatccttcattgaacagttttagcattgctgaagatgaagagtatgttattccaacactactcgctgccaaacaggttaatccaaacttgaagatcgttgcttctccatggtcacctcctgcttggatgaaaacatcaaatagcttagacggtggatatttcaattcagattacacagacatctacggaaactattgggtaa

Contig 319-midgut trypsin

Tttttttttcatactttttattttgtttattctataaataacttattttactccgtcaacttacagtaaaacaaaaacaagttgatttctttattttacatgcattgcagacacacgtatttacattttgcacgcttctgttctaaggaatttcaattcctgatgtttcggagatccagtccaaatatgaagtaactcgtgtaaatccatttggagctatatctgggcaatatccagcaccatacgaatcaattcctatttgtatgcttgaatccccatcttgtacaaacaatggactcccactgtcaccactgcagatactggtgtctccattgccaaaagcacacacttgtgtatctggaacatcgccccaactctccgcgcattcttcgttggtcagtattgttgtttcaacccactttagtagatcatcatttgaagagtcatcgtcgtcgtctccaccccatccggcagctgtagctctttgtcctgcaaaagactcatcttgctgagagcgagaaggtaaactgataggttgaattacgtctgaatattgcactctctcagaaagttttataagggctaagtcgttctctactgttacatcattatagtccggatgcacgaaccgagaatttgatgacacctcaactcttccttcctcattttgatcctccggattaagagctccaagaacaattgtgaactcatttaaaccgtcaacagcacagtgagctgcagtcaaaacccattcttcattgagaagactagaaccgcataaggctacccatccgtctcctagctcatggtacaggcgagcaaggaatggaaattggccacgggtcgccaactgacctccagctatcctgccccattgtatgaagttttcatatccttttatcgtgctgttacgatgtactttaaatacaggcatacgaggagcagcctgagccgccacagcgcaaatcaacaaaagtaggaacgttctcatcttcgttattagcgcg

Contig 321-actin 5C isoform B

cattactttcattattatttggaatttccgaacttttttatacaatacaaactttataatttttaagcattttcctttttcacttttaaatattcttattttaacattgagcatggaatgcatgttctttaaccaagcttcttgttataaacttacagaaacccgggaaaaatcagatctcgatacaggcagaaaccctggaaaatactcagatctcaaaacaggcaacttaacttccttgtatgcgttatttcacactaacaatcctttataatacacgctgactaaacgtttcatgatagagaaaagtctgtatcaaaagtgatgcctgggtttgtccaacagggcttctatccagaggaacgggttgcaaaaactttacaaacttccagcttagaagcatttcctgtgcacgatggatggtccagactcgtcgtattcttgtttgctaatccacatctgttggaacgtagacagggaagctaagatggagccaccaatccatacagagtacttcctctcggggggagcgattatcttgatcttcatggtggaaggagccagggcagtaatctccttctgcatcctatcagcgatgcctgggtacattgtggtgcctccagacagaacggtgtttgcgtacaggtcctttcggatatccacgtcgcacttcatgatagagttgtaggtggtttcatggattccgcacgcttccataccaaggaacgaaggttggaacaaggcctcggggcacctaaacctttcatttccaatagttataacttgaccatcaggcaattcataggatttttcaagggaactagaggaagccgcggtggccatttcttgctcgaagtctagggctacatagcaaagtttttccttgatgtctcggacgatttctcgctcggccgtggtagtgaaactgtagcctctctcggtaaggatcttcatgaggtagtccgtcaggtctcggccagccaagtccagacgcaagatggcgtggggcaatgcataaccttcatagattggcacagtgtgagagacaccatctccggagtcgagcacaataccagttgttctaccagaagcatacaatgacagcacagcctgaattgctacatacatggcaggggtgttgaaagtctcgaacatgatctgtgtcatcttttctctgttggctttggggttgagtggggcttccgtaagaagaacagggtgctcctcgggcgccactcgaagttcattgtagaatgtgtgatgccagattttctccatgtcatcccaattagtaacaataccatgttcaattggatatttcaaagttagaatacctcgcttagactgggcctcatcacctacataactgtccttttgacccattcctaccattacaccctgatgtcttggtcgaccaacaattgatgggaaaactgctctgggtgcatcatcgcctgcaaacccagctttgcacattccggatccattgtccactacaagggcagcaacttcttcgtcacacatggttagtatttaattctctacacagacgtatcactaacagtacacagcaaactgtacaaatctgtcgcaagtgaccgaacgaaccgtttagggaaac
